# Enzyme-free digital counting of endogenous circular RNA molecules in B-cell malignancies

**DOI:** 10.1038/s41374-018-0108-6

**Published:** 2018-08-07

**Authors:** Mette Dahl, Iben Daugaard, Maria Schertz Andersen, Thomas Birkballe Hansen, Kirsten Grønbæk, Jørgen Kjems, Lasse Sommer Kristensen

**Affiliations:** 1 Department of Hematology, Rigshospitalet, Copenhagen University Hospital, DK-2100 Copenhagen, Denmark; 2 Biotech Research and Innovation Centre, BRIC, Copenhagen University, DK-2100 Copenhagen, Denmark; 3 Department of Molecular Biology and Genetics (MBG), Aarhus University, DK-8000 Aarhus, Denmark; 4 Interdisciplinary Nanoscience Center (iNANO), Aarhus University, DK-8000 Aarhus, Denmark

## Abstract

Circular RNAs (circRNAs) are covalently closed endogenous molecules with tissue- and disease-specific expression patterns, which have potential as diagnostic and prognostic biomarkers in cancer. The molecules are formed by a backsplicing event linking the 3′-end of an exon to the 5′-end of the same or an upstream exon, and they exert diverse regulatory functions important in carcinogenesis. The landscape of circRNA expression has not been characterized in B-cell malignancies, and current methods for circRNA quantification have several limitations that prevent development of clinically applicable assays. Here, we demonstrate that circRNAs can be accurately quantified without enzymatic reactions or bias using color-coded probes (NanoString technology). First, we performed high-throughput RNA sequencing (RNA-seq) of several mantle cell lymphoma and multiple myeloma cell lines to profile the genome-wide landscape of circRNA expression. We detected several circRNAs known to be deregulated in other cancers and identified a novel circRNA from the *IKZF3* gene. Based on these data, we selected 52 unique circRNAs for which we designed color-coded probes spanning their specific backsplicing junctions. These circRNAs were quantified in cell lines and patient samples from several different B-cell malignancies (mantle cell lymphoma, multiple myeloma, follicular lymphoma, diffuse large B-cell lymphoma, Burkitt lymphoma and chronic lymphocytic leukemia) simultaneously using the NanoString technology. The circRNA expression profiles obtained could distinguish different B-cell malignancies, and confirmed the presence of the novel circRNA derived from *IKZF3*. The NanoString assays were specific for circRNA detection and data were more reproducible and quantitatively more accurate than RNA-seq data. In addition, we obtained high-quality data on severely degraded RNA samples from formalin-fixed, paraffin-embedded (FFPE) tissues. Together, we provide a map of circRNA expression in B-cell malignancies and present an enzyme-free digital counting methodology, which has the potential to become a new gold standard for circRNA quantification.

## Abstract (web summary)

Endogenous circular RNA (circRNA) expression has not been characterized in B-cell malignancies, and current methods for circRNA quantification have several limitations. Here, the authors provide a map of circRNA expression in B-cell malignancies based on high-throughput RNA sequencing and present an enzyme-free digital counting methodology, which has the potential to become the gold standard for circRNA quantification.

## Introduction

Circular RNA (circRNA) is a recently discovered component of the non-coding genome adding yet another layer of complexity to the gene regulation involved in cell differentiation and homeostasis, as well as in the development of various diseases such as cancer [1, 2]. CircRNAs are formed by a backsplicing event covalently linking the 3′-end of an exon to the 5′-end of the same or an upstream exon [3]. Most circRNAs originate from a host gene and their biogenesis is promoted by flanking homologous inverted repeats (*Alu* repeats in humans) bringing the splice sites in close proximity. A common feature of circRNAs is their high stability owing to the lack of free ends, which renders them resistant to exonucleolytic degradation [4], and many of their diverse functions are directly related to this, including sponging of miRNAs [5–7] and protein scaffolding [8, 9]. CircRNAs may also regulate host gene expression either directly by functioning as transcriptional enhancers or indirectly by competing with linear splicing [10, 11]. Others function as global or specific regulators of protein translation [12, 13], and some even function as templates for translation [14–16].

A number of studies have reported differential expression of specific circRNAs in various tumor tissues, and due to the high stability of these molecules they are promising biomarkers in cancer diagnostics [1]. However, knowledge on circRNA expression and function in hematologic malignancies is sparse [3, 17, 18], and these molecules have not previously been studied in B-cell malignancies. These diseases arise at various stages of B-cell development, maturation and differentiation, most from B-cells in the germinal center (GC) [19], whereas chronic lymphocytic leukemia (CLL) is thought to arise from memory B-cells in peripheral blood [20]. The majority of mantle cell lymphomas (MCLs) arise from naive B-cells before they enter the GC [21], and multiple myeloma (MM) develops from memory B-cells or plasmablasts that migrate to the bone marrow and differentiate into malignant plasma cells [22].

Several methodological obstacles impair the detection and quantification of circRNAs [1, 23]. First, circRNAs lack poly(A) tails and are therefore discarded during library preparation for high-throughput RNA sequencing (RNA-seq) when using protocols that rely on a poly(A) purification step for removal of ribosomal RNA (rRNA). Thus, most publically available RNA-seq data sets, including the majority of data from The Cancer Genome Atlas (TCGA), cannot be analyzed for circRNA expression. Second, it is necessary to actively search for circRNAs as sequencing reads mapping to the backsplicing junctions of circRNAs are discarded in standard bioinformatics algorithms, because they do not map to the linear reference genome. Similarly, conventional reverse transcription-quantitative PCR (RT-qPCR) assays do not distinguish circular from linear RNA when using the linear genome as template for primer design. Even when actively searching for circRNAs, methodological challenges like template switching and rolling circle amplification during RT and amplification bias during PCR may hamper the results [1, 23, 24]. Therefore, the circular nature of the transcripts needs to be validated. A commonly used validation method utilizes the fact that circRNAs are generally resistant to degradation by RNase R, an exonuclease, which degrades linear RNA. However, variability between individual RNase R treatments has been observed [4], and some circRNAs seem to be sensitive to RNase R [25]. Because of these problems, northern blotting, which does not rely on RT and PCR amplification, is currently regarded as the gold standard for circRNA detection. However, this method requires large quantities of RNA, is labor intensive and not quantitatively accurate. Taken together, no current methods have the capability of accurately quantifying circRNAs and there is no consensus on how to evaluate or account for the possible bias introduced during RT and PCR amplification of RNA-seq and RT-qPCR protocols.

Intriguingly, a relatively novel digital counting technology, termed NanoString, is completely free of any enzymatic reactions and accurate for quantifying linear mRNAs [26]. The technology is based on a dual-probe hybridization using a biotinylated capture probe and a unique color-coded reporter probe. A combination of fluorophores on the reporter probe provides a unique barcode for each target, allowing for multiplexing of up to 800 targets in one experiment [27]. Because the probes only target short RNA-sequences and no RT or amplification steps are involved, this method is particularly suitable for analyzing highly degraded RNA isolated from formalin-fixed paraffin-embedded (FFPE) tissues, which are often routinely stored in pathology departments along with patient data [26, 28]. However, the NanoString technology has not previously been used for the detection of circRNA.

In this study, we profiled the genome-wide landscape of circRNA expression in MCL and MM cell lines using RNA-seq. Based on these data, we designed a panel of NanoString assays for 52 unique circRNAs to analyze on the nCounter *SPRINT*^TM^ Profiler from NanoString Technologies. To investigate the potential of this technology for circRNA detection and quantification, we analyzed high-quality RNA samples and RNase R-treated samples from the MCL cell lines, as well as low-quality RNA derived from the same cell lines after formalin fixating and paraffin embedding the cells. In addition, we analyzed RNA isolated from several different cell lines from B-cell malignancies, as well as paired fresh frozen and FFPE patient samples. Finally, we compared the NanoString data with RNA-seq- and RT-qPCR data.

## Materials and methods

### Cell lines, patient samples and ethical considerations

MCL cell lines (REC-1 and UPN2), diffuse large B-cell lymphoma (DLBCL) cell lines (HT, OCI-Ly3, Toledo and U2932), Burkitt lymphoma (BL) cell lines (Raji and Ramos) and MM cell lines (RPMI-8826 and OPM2) were cultured in RPMI-1640 with 10% fetal bovine serum (FBS). The MM cell line (MOLP2) was cultured in PRMI-1640 with 20% FBS. The MCL cell lines (Z138 and Granta-519) were cultured in Iscove’s modified Dulbecco’s medium (IMDM) with 10% Horse Serum and in Dulbecco’s modified Eagle’s medium (DMEM) with 10% FBS, respectively. The MM cell line (NCI-H929) was cultured in RPMI-1640 with 1 mM sodium pyruvate and 50 µM 2-mercaptoethanol and 10% FBS, whereas the MM cell line (JJN3) was cultured in a medium containing 40% IMDM, 40% DMEM and 20% FBS.

Paired fresh frozen and FFPE tissue samples from archived lymph nodes were obtained from five patients with MCL, three patients with CLL, three patients with follicular lymphoma (FL) grade 1–2 and two patients with DLBCL. Specimens were collected between 1990 and 2008 at the Department of Pathology, Rigshospitalet, and patients were diagnosed according to the WHO lymphoma classification.

This study was performed in accordance with the Declaration of Helsinki and Danish legislation.

### Formalin-fixation and paraffin embedding of cell lines

Formalin-fixation and paraffin embedding of cells was carried out on a Peloris tissue processor (Leica Biosystems, Wetzlar, Germany) with a fixation time of approximately 12 h after assembly of cell pellets by addition of human plasma and bovine thrombin (BIOFAC, Kastrup, Denmark) along with a Mayer’s hematoxylin staining for visualization.

### RNA isolation and integrity assessment

RNA from cell lines was isolated using the Allprep DNA/RNA/miRNA universal kit (Qiagen, Hilden, Germany). For RNA isolation of FFPE cell lines, the Allprep DNA/RNA FFPE kit (Qiagen) was used following a xylene deparaffinization. RNA from fresh frozen patient samples was isolated with the RNeasy mini kit (Qiagen). Tissues were disrupted using the rotor-stator homogenizer Dispomix (Xiril, Hombrechtikon, Switzerland). RNA from the FFPE patient samples was isolated from two freshly cut sections of 20 µm for each sample using the RNeasy FFPE kit (Qiagen) following a xylene deparaffinization. Regardless of the kit used, on-column DNase (Qiagen) treatment was performed.

RNA concentrations were assessed using a Nanodrop2000c (Thermo Fisher Scientific, Waltham, MA, USA) instrument and RNA integrity number (RIN) value was measured on the Bioanalyzer 2100 (Agilent Technologies, Santa Clara, CA, USA) using the RNA Nano 6000 kit (Agilent Technologies). Representative results are shown in Supplementary Figure 1 and RIN values are listed in Supplementary Table 1.

### High-throughput RNA-seq

One microgram of total RNA was rRNA depleted using the Ribo-Zero rRNA Removal Kit (Human, Mouse, Rat) (Epicentre, Madison, WI, USA) followed by a purification step using AMPure XP Beads (Beckman Coulter, Brea, CA, USA). Sequencing libraries were generated using the ScriptSeq v2 RNA-Seq Library Preparation Kit (Epicentre) using 12 PCR cycles for amplification. Purification was performed using AMPure XP Beads (Beckman Coulter). The final libraries were quality controlled on the 2100 Bioanalyzer (Agilent Technologies) and quantified using the KAPA library quantification kit (Kapa Biosystems, Wilmington, MA, USA). RNA-seq was performed on the HiSeq 4000 system (Illumina, San Diego, CA, USA) at the Beijing Genomics Institute (BGI) using the 100 paired-end sequencing protocol with nine samples pooled on one lane.

### RNA-seq data analysis

Sequencing data were quality controlled (Phred score 20) and adapter trimmed using Trim Galore. Filtered data were mapped to the human genome (HG19) using TopHat2. CircRNA expression was quantified based on a stringent version of the find_circ bioinformatics algorithm [29]. Reads per million (RPM) refers to sequencing reads aligning across the particular backsplicing junction normalized to the total number of raw reads. Circular-to-linear (CTL) ratios were defined as the number of reads spanning the backsplicing junction divided by the average number of linear reads spanning the splice donor- or splice acceptor sites of the backsplicing junction. In addition, we analyzed the raw RNA-seq data using another bioinformatics algorithm known as CIRI2 as described previously [30].

### Data availability

Raw and processed RNA-seq data have been deposited in the Gene Expression Omnibus (GEO) database under accession number [GSE108111].

### Sanger sequencing across backsplicing junctions of selected circRNAs

Complementary DNA (cDNA) synthesis was performed on 500 ng total RNA from the MM cell line, NCI-H929, using the M-MLV reverse transcriptase (Thermo Fisher Scientific) and random primers. The cDNA was diluted fivefold in PCR grade water and used as template for PCR. The reaction mixtures consisted of 4 µL template in a total volume of 25 µL using a 1 × final concentration of the Taq Reaction buffer, 500 µM dNTPs, 1 unit *Taq* DNA Polymerase, recombinant and a final MgCl_2_ concentration of 1.5 mM. Primers (Supplementary Table 2) were used at a final concentration of 300 nM. The cycling protocol was initiated by one cycle at 94 °C for 3 min, followed by 40 PCR cycles at 94 °C for 20 s, 60 °C for 20 s, and 72 °C for 30 sec and a final extension step of 72 °C for 7 min. Five µL of each PCR product was loaded on 2% agarose gels stained with SYBR™ Safe DNA Gel Stain (Thermo Fisher Scientific) and visualized under UV light after electrophoresis. The remaining 15 µL of each PCR product was cleaned up using the QIAquick PCR Purification Kit (Qiagen) and Sanger sequenced in both forward and reverse directions using the service of GATC (GATC Biotech, Konstanz, Germany).

### Northern blotting

Ten µg of high-quality RNA from the NCI-H929 cell line was treated with 10U RNase R (Epicentre) or mock treated for 10 min at 37 °C and loaded on a 1.2% agarose gel. The gel was run for 2.5 h and RNA was transferred to an Amersham™ Hybond™-N + membrane (GE Healthcare). After transfer, the membrane was hybridized with [32 P]-labeled probes at 55 °C. The membranes were exposed to a phosphoscreen for 48 h and analyzed in Image Lab™ (Bio Rad). Probe sequences are listed in Supplementary Table 2. Due to the small size of circIKZF3, we only designed one probe targeting its backsplice junction.

### NanoString nCounter codeset design and circRNA expression analyses

A custom CodeSet of capture and reporter probes was designed to target regions of 100 nucleotides overlaying the backsplicing junctions of 52 unique circRNAs, each probe with a target sequence of exactly 50 nucleotides (Supplementary Table 3). In addition, five linear reference genes were included. The circRNA targets were selected based on the RNA-seq data from the MM and MCL cell lines and included both highly and lowly expressed circRNAs. Two hundred ng and 400 ng of low- and high-quality RNA, respectively, were subjected to nCounter™ *SPRINT* (NanoString Technologies, Seattle, WA, USA) analysis according to the manufacturer’s instructions. When analyzing the data using the nSOLVER 3.0 software (NanoString Technologies), background subtraction was performed using the mean of negative controls, and the geometric mean of positive controls was used for normalization. A second normalization using the geometric mean of the four most stable linear reference genes (*ACTB*, *PUM1*, *SF3A1* and *UBC*) was performed. The reference genes have previously been shown to be stably expressed in B-cell malignancies [31, 32].

### RNase R experiments

Five µg of RNA was either treated with 5U RNase R (Epicentre) or mock treated. The RNA samples were denatured for 30 s at 95 °C followed by the addition of a master mix consisting of RNase R (or nuclease-free water for the mock treated samples), 1 × final concentration of reaction buffer and RiboLock (Thermo Fischer Scientific). Reactions were incubated for 10 min at 37 °C. Following RNase R or mock treatment, each sample was diluted to a total volume of 300 µL with nuclease-free water, washed with one volume of ethanol (96–100%) and applied to an RNeasy mini spin column (Qiagen) and centrifuged for 15 s at 10,000 *g*. Subsequently, two washing steps with 500 µL buffer RPE were performed, and an additional 2-min centrifugation step was carried out before eluting the samples in 50 µL nuclease-free water. RNA concentrations were standardized before NanoString analyses.

### RT-qPCR analyses

cDNA synthesis was done in duplicate with 1 µg of RNA from each MCL cell line using the M-MLV Superscript III reverse transcriptase (Invitrogen, Carlsbad, CA, USA) and random primers. The cDNA was diluted fivefold and used as template for qPCR. The reaction mixtures consisted of 2 µL template in a total volume of 20 µL using a 1 × final concentration of the 2 × SYBR Green I Master (Roche, Basel, Switzerland) and primers (Supplementary Table 4) at a final concentration of 500 nM. The cycling protocol was initiated by 1 cycle at 95 °C for 10 min, followed by 40 PCR cycles at 95 °C for 10 s, 60 °C for 20 s, and 72 °C for 20 s and a melt curve analysis. Each primer pair was evaluated using serial dilutions, and experiments were performed according to the MIQE guidelines [33]. The PCR efficiency (*E*) and quantitative accuracy (Pearson’s correlation coefficients) for each assay are listed in Supplementary Table 5. The RT-qPCR data were normalized using the same four reference genes as used to normalize the NanoString data.

### Heat maps and hierarchical cluster analyses

For the heat maps and hierarchical cluster analyses, a z-score transformation of the normalized counts for each circRNA was performed. Clustering was done with the Pearson’s correlation distance metric and the linkage method called average using the nSOLVER 3.0 software (NanoString Technologies).

### Statistical analyses

All statistical tests were performed using Prism 7 (GraphPad, La Jolla, CA, USA). The reproducibility between RNA-seq- and NanoString data, RT-qPCR and NanoString data, as well as NanoString intra-assay reproducibility, was evaluated using linear regression. Analyses of 2 × 2 tables were done using Fisher’s exact tests. All *P*-values were two-tailed and considered significant if < 0.05.

## Results

### RNA-seq profiling reveals high expression of circRNAs in MCL and MM

**Fig. 1:**
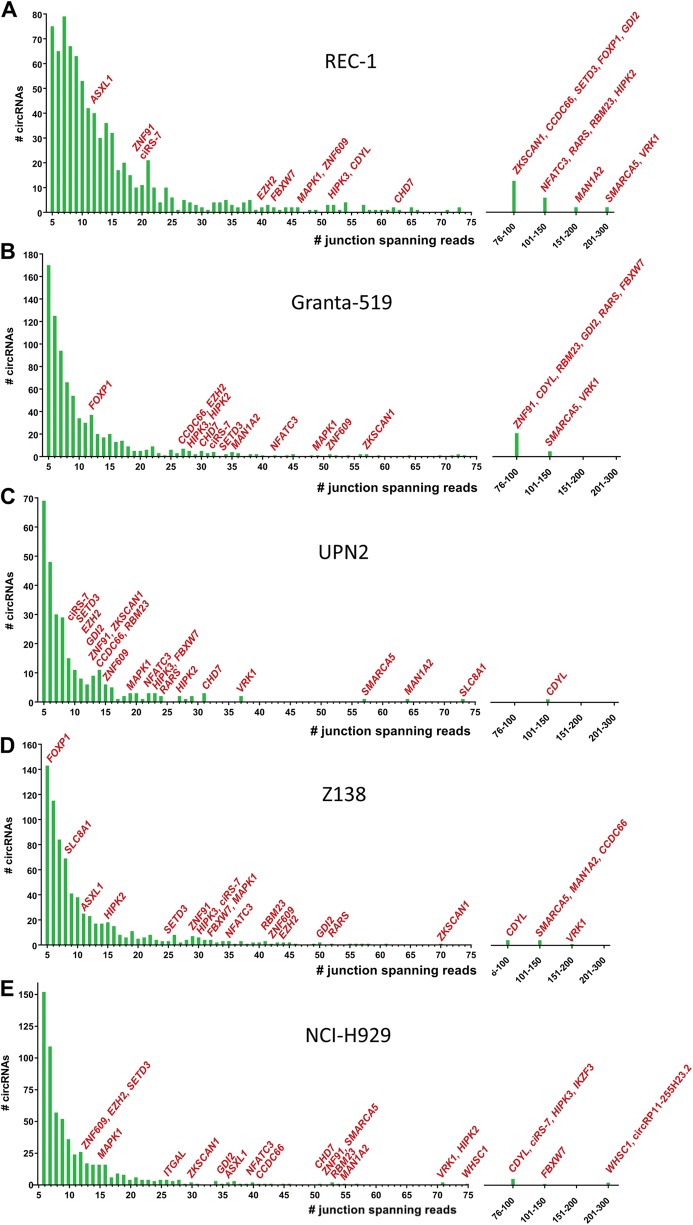
CircRNAs are highly abundant in MM and MCL cell lines. **a**-**e** CircRNA species in the cell lines REC-1 **a**, Granta-519 **b**, UPN2 **c,** Z138 **d** and NCI-H929 **e** supported by at least five reads are displayed. The number of circRNAs (*y* axis) detected by a specific number of reads in RNA-seq (*x* axis) is shown. Host genes of circRNA species of particular interest are indicated

RNA-seq profiling of four different MCL cell lines, REC-1, Granta-519, UPN2, Z138 and the MM cell line, NCI-H929, revealed 813, 816, 741, 279 and 619 unique circRNAs, respectively, all supported by at least five backsplice junction-spanning reads using the find_circ bioinformatics algorithm (Fig. 1). CircRNAs composed of two exons were most frequent in all cell lines (Supplementary Figure 2). The highest percentage (12.4%) of circRNAs, with expression levels higher than their respective host genes, was observed in Z138, whereas the lowest percentage (7.5%) was observed in Granta-519 (Supplementary Figure 2). Host genes producing a single circRNA were most frequent in all cell lines. However, some genes produced several unique circRNAs, including *ATM*, *XPO1* and *WHSC1*, which are genes involved in lymphomagenesis (Supplementary Figure 2).

**Fig. 2:**
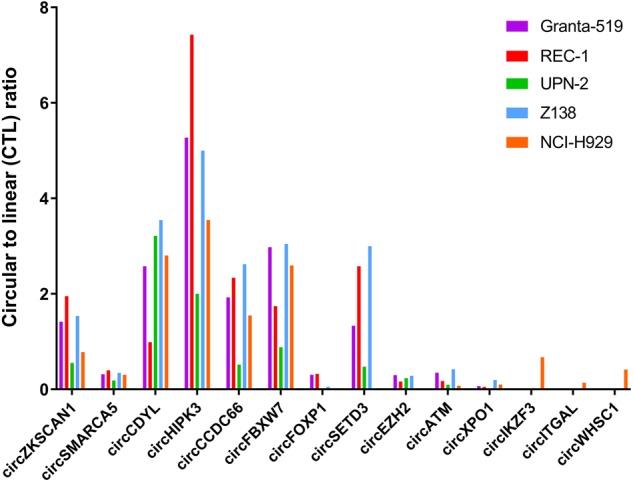
Barplot showing the circular-to-linear (CTL) ratios of 14 different cancer-related circRNAs in four different mantle cell lymphoma (MCL) cell lines and one multiple myeloma (MM) cell line (NCI-H929) color-coded as indicated. Several of these circRNAs were expressed at a higher level than the corresponding linear host genes (CTL ratio > 1) in at least one of the cell lines studied. For ciRS-7, there was no linear reads spanning the splice donor and splice acceptor sites of the backsplicing junction. Thus, no CTL ratios could be calculated for this particular circRNA

Interestingly, we found several circRNAs, which have previously been implicated in cancer, including ciRS-7 [34–36], circHIPK3 [7, 37], circCCDC66 [38], circFBXW7 [16], circSMARCA5 [39, 40], circCDYL [37] and circZKSCAN1 [41]. We also detected circRNAs from genes involved in lymphomagenesis and the development of MM, including *FOXP1* [42], *SETD3* [43], *EZH2* [44], *ATM* [45], *XPO1* [46], *IKZF3* [47], *CD11A (ITGAL)* [48] and *WHSC1* (*MMSET*) [49]. The circRNA derived from *IKZF3* is not listed in circBase [50] and has, to our knowledge, not previously been reported. Several of these circRNAs were expressed at higher levels than the corresponding linear host genes (Fig. 2).

**Fig. 3:**
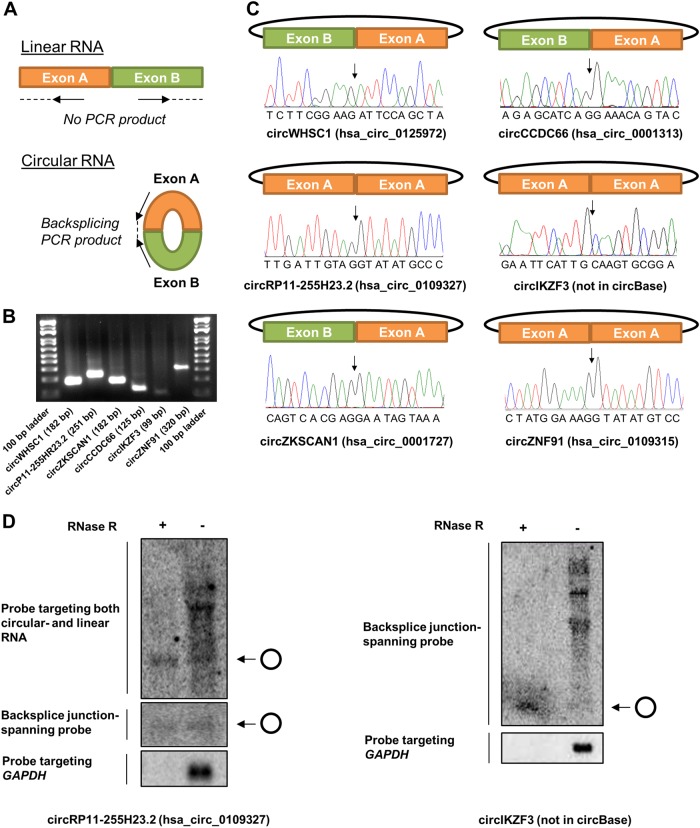
Validation of RNA-seq data for selected circRNAs using PCR with divergent primers and Sanger sequencing. **a** Schematic representation of divergent PCR primer design that ensures circular RNA-specific amplification. **b** Agarose gel electrophoresis of circRNA-specific PCR on six different circRNAs as shown. The expected amplicon size is denoted in parenthesis, and 100-bp molecular marker is included on both sides of the gel. **c** For all six circRNAs analyzed, Sanger sequencing chromatograms across the backsplicing junction are shown. Arrows indicate the exon-exon junctions. **d** Northern blotting for circRP11-255H23.2 and circIKZF3. Due to the small size of circIKZF3, we only designed one probe targeting its backsplice junction

The RNA-seq data were validated for several of the circRNAs, including the novel circRNA from *IKZF3*, by RT-PCR with divergent primers (Fig. 3a, b) and Sanger sequencing across the backsplicing junctions (Fig. 3c). Northern blotting was also successfully performed for the novel circRNA from *IKZF3* and for the second highest expressed circRNA in NCI-H929, circRP11-255H23.2, which has not previously been confirmed by northern blotting (Fig. 3d).

### CircRNA expression profiles in cell lines from various B-cell malignancies

**Fig. 4:**
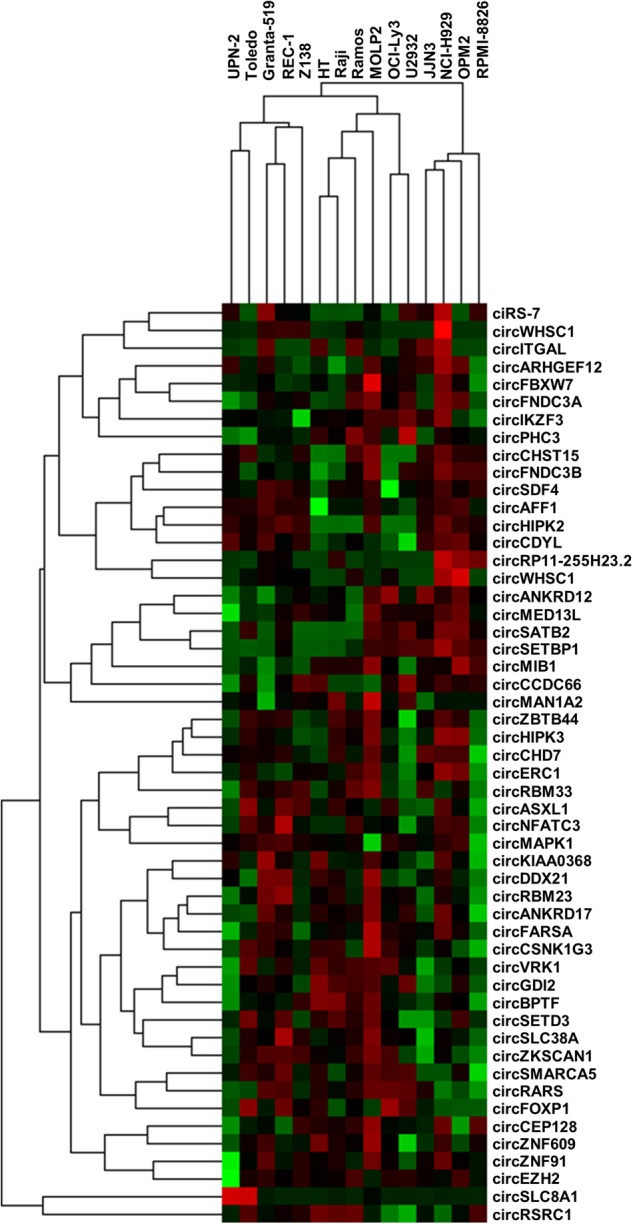
Hierarchical cluster analysis of NanoString data from 15 malignant B-cell lines. Heatmap showing expression and clustering of 52 circRNAs (rows) from 15 malignant B-cell lines (columns). The cell lines comprise MM cell lines (JJN3, NCI-H929, OPM2, RPMI-8826 and MOLP2), MCL cell lines (UPN2, Granta-519, REC-1 and Z138), BL cell lines (Raji and Ramos), DLBCL cell lines, ABC-type (OCI-Ly3 and U2932) and DLBCL cell lines, GCB-type (HT and Toledo)

Based on the RNA-seq data, we designed NanoString assays targeting 52 circRNA candidates, mainly focusing on circRNAs previously implicated in other cancers and circRNAs produced from host genes involved in lymphomagenesis. These assays were used to elucidate the expression of circRNA in B-cell malignancies by analyzing RNA from 15 different cell lines, including four DLBCL, two BL, four MCL and five MM. Four of the five MM cell lines clustered separately from all the other cell lines, whereas one (MOLP2) clustered together with the DLBCL cell line of GCB-type (HT) and the two BL cell lines (Raji and Ramos). MOLP2 is the only MM cell line expressing IgD, indicating origin from a GC B-cell, which is also the origin of BL and DLBCL of the GCB-type. In addition, the DLBCL cell lines of ABC-type (OCI-Ly3 and U2932) clustered together, and the four MCL cell lines clustered together. The novel circRNA from *IKZF3* was highly expressed in NCI-H929 (confirming the RNA-seq data) and also relatively abundant in MOLP2 and in OCI-Ly3 and U2932 (Fig. 4).

### The nanostring assays are specific for circRNA detection

**Fig. 5:**
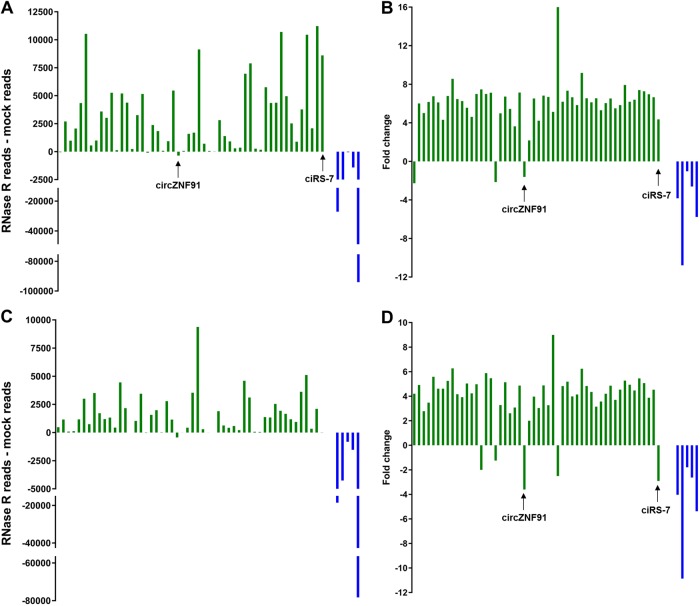
NanoString analysis on RNase R-treated samples. **a**, **c** Barplot depicting relative raw counts obtained from NanoString analysis of 52 circRNAs (green) and five linear control mRNAs (blue) in mock and RNAse R-treated samples from cell lines Granta-519 **a** and Z138 **c**. **b**, **d** From **a** and **c**, the fold-change (RNAse R treated/mock treated) is shown for transcripts for which RNAse R treated > mock treated, whereas (mock treated/RNAse R treated)*(−1) is shown for transcripts for which RNAse R treated < mock treated

RNase R experiments were performed to investigate whether the NanoString assays were specific for circRNA detection. RNA samples from two MCL cell lines (Granta-519 and Z138) were treated with RNase R or mock treated. Among the 52 circRNAs analyzed, only three and five species were not enriched by more than twofold upon RNase R treatment in Granta-519 and Z138, respectively. (Fig. 5). Unexpressed circRNAs are not expected to be enriched. As an example, ciRS-7 was clearly enriched in Granta-519 where it is highly expressed, but not in Z138, where it is expressed at a very low level. The only circRNA that was expressed in both cell lines and clearly not resistant to RNase R was circZNF91. The five linear RNAs analyzed by NanoString were degraded to various extents as expected. Overall, an average increase in circRNA expression of approximately sixfold was observed for the circRNAs, whereas the linear RNAs were on average decreased by approximately fourfold.

### The nanostring assays provide reproducible data

To test the reproducibility of the NanoString technology for circRNA detection, two RNA samples of high and low quality in terms of RNA degradation were analyzed twice on different days. A high correlation between the NanoString data from individual replicates were observed for both high- and low-quality RNA samples (Supplementary Figure 3).

### Correlation between nanostring data from high- and low-quality RNA

To investigate how well data from samples of high quality compare with data from samples of low quality, we formalin-fixed and paraffin-embedded cells from each of the four MCL cell lines. Quality assessments confirmed that RNA from the FFPE cells was degraded (Supplementary Figure 1). Nevertheless, a good correlation between the NanoString data from high- and low-quality RNA was observed for all four cell lines (Supplementary Figure 4). In addition, unsupervised hierarchical cluster analyses clearly separated the samples according to cell line and not according to RNA quality (Supplementary Figure 5).

### Correlation between nanostring data from paired fresh frozen and FFPE patient samples

NanoString data from paired fresh frozen and FFPE tissue samples from patients with various B-cell malignancies were analyzed to investigate how well the technology performs using old archival FFPE tissue samples. We calculated DV 200 values, a measure of the percentage of RNA fragments above 200 nucleotides, to analyze the RNA quality of the samples (Supplementary Table 1). Four FFPE samples were severely degraded (DV 200 < 0.33), whereas the remaining FFPE samples had DV 200 values above 0.40. Despite the poor RNA quality, a relatively high correlation between the NanoString data from high- and low-quality RNA was observed for all sample pairs (Supplementary Figure 6), with the exception of one pair (patient 22), for which a normalization flag in the NanoString analyses indicated that the data was of poor quality. This sample pair was therefore removed from further analyses. Data from the FFPE samples with DV 200 values above 0.40 correlated better with data from their respective paired fresh frozen sample (mean *R*^2^ value: 0.85, range: 0.70–0.97) compared with sample pairs for which the FFPE sample had a DV 200 value below 0.33 (mean *R*^2^ value: 0.69, range: 0.57–0.83). Finally, unsupervised hierarchical cluster analyses, using samples with DV 200 values above 0.40, clearly separated the samples according to individual patients and not according to RNA quality (Supplementary Figure 7).

### Comparison of nanostring data with RNA-seq- and RT-qPCR data

**Fig. 6:**
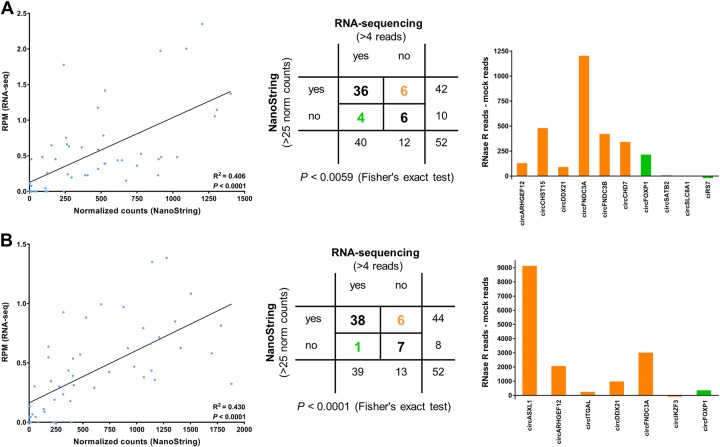
Comparison of circRNA expression data by NanoString and RNA-seq for the cell lines where RNase R experiments were performed. **a, b** Correlation between RPM and NanoString-derived normalized counts from Z138 **a** and Granta-519 **b** cell lines with corresponding linear regression statistics and R-squared values (left panel). Middle panel shows 2 × 2 contingency table using pre-defined expression stratification as shown. NanoString- or RNA-seq-specific detection is color-coded orange or green, respectively. RNAse R sensitivity for the NanoString (orange bars) or RNA-seq-specific circRNAs (green bars) are shown to the right by relative counts between mock and RNAse R-treated samples for Z138 **a** and Granta-519 **b**. circASXL1 was an extreme outlier and removed from the analyses

Highly significant correlations between Nanostring- and RNA-seq data (analyzed by find_circ) were observed (Fig. 6, left panel and Supplementary Figure 8, left panel). However, a bias was observed whereby the expression of specific circRNAs was systematically estimated to be either higher or lower by RNA-seq relative to NanoString in all cell lines. These circRNAs included circCDYL, circVRK1, circSMARCA5, circCCDC66, circZKSCAN1 and circHIPK3 (Supplementary Figure 9). Therefore, to examine their expression by a third method, we designed RT-qPCR assays for these circRNAs and analyzed the MCL cell lines. For all, except circVRK1, we observed a good correlation between NanoString- and RT-qPCR data with no systematic over- or underestimations of the expression levels (Supplementary Figure 10). To investigate if the systematic differences between RNA-seq and NanoString relate to the bioinformatics algorithm used to analyze the RNA-seq data, we also quantified circRNA expression data using CIRI2 [30]. The correlations between find_circ and CIRI2 were not perfect, but overall better than observed when comparing NanoString- and find_circ data. Therefore, the selection of bioinformatic algorithm does not appear to explain the majority of the bias. However, circVRK1 was consistently estimated to be lower expressed by CIRI2 compared with find_circ (Supplementary Figure 11). Thus, the CIRI2 data was in better agreement with the NanoString data than find_circ for this particular circRNA.

We also analyzed the NanoString- and RNA-seq data (by find_circ) as dichotomous variables, using pre-defined cut-offs for grouping the circRNAs into expressed or not expressed. We observed a significant correlation between the data for all cell lines (Fig. 6, right panel and Supplementary Figure 8, right panel). To further analyze the circRNAs for which RNA-seq and NanoString data differed, we used the results from the RNase R experiments on Z138 and Granta-519. In Z138, six circRNAs were detected only by NanoString, all of which were enriched in the RNase R experiment (defined as an increase of >50 raw counts), and four were detected only by RNA-seq, of which only one (circFOXP1) was enriched in the RNase R experiment (Fig. 6a, right panel). In Granta-519, six circRNAs were detected only by NanoString and one was detected only by RNA-seq. Five of the six circRNAs detected only by NanoString were enriched in the RNase R experiment. This also applied to the one circRNA (circFOXP1) only detected by RNA-seq (Fig. 6b, right panel). RT-qPCR confirmed that circFOXP1 is expressed at low levels in both Granta-519 and Z138 (Supplementary Figure 12), and despite being below the arbitrary cut-off, it was indeed expressed above background in the NanoString data (data not shown). RT-qPCR also confirmed that circASXL1 and circCHD7 are expressed in Granta-519 and Z138, respectively, although not being detected by find_circ. These two circRNAs were also detected by CIRI2. In addition, circSLC8A1, which was detected by find_circ and not by NanoString in Z138, could not be detected by RT-qPCR in this cell line (Supplementary Figure 12).

Finally, for some of the RT-qPCR assays we observed concatemers, which are likely formed by rolling circle amplification during the RT step. This applied to the assays targeting circZKSCAN1, circSMARCA5, circASXL1 and circFOXP1. For circFOXP1, melting curve analysis revealed two peaks at 77 °C and 85 °C, respectively. These corresponded to two products detected using gel electrophoresis, namely the expected amplicon of 49 bp and a product of approximately 600 bp. Because the spliced length of circFOXP1 is 587 bp, the second product is likely to represent a concatemer resulting from rolling circle amplification (Supplementary Figure 13).

## Discussion

Reliable detection and quantification of circRNAs is currently hampered by the risk of introducing experimental bias and artifacts as most available methods, including RNA-seq, microarray and RT-qPCR, employ RT and/or PCR amplification. For this reason, there is a high demand for novel reliable methods for accurate quantification of circRNAs.

The NanoString technology is enzyme-free and, therefore, not prone to artifacts and bias associated with RT and PCR amplification. It is a digital counting method, which allow up to 800 targets to be investigated simultaneously with minimal hands-on-time. Following over-night hybridization, results can be obtained within approximately 6 h and the downstream analyses do not require bioinformatic expertise and can be completed on a standard laptop. For these reasons, the NanoString technology is easily implemented in a clinical setting and is already used clinically for breast cancer prognostication based on a 50-gene mRNA test [51], which works well on RNA isolated from FFPE tissue sections [52].

The expression of circRNA molecules in B-cell malignancies has not previously been explored and there is a lack of reliable high-throughput methodologies for investigating circRNA expression profiles in low-quality RNA samples from FFPE tissues. Therefore, we decided to explore the potential of the NanoString technology for quantification of circRNA molecules in both high- and low-quality RNA samples from malignant cell lines and samples from patients with B-cell malignancies.

First, we performed RNA-seq of MM and MCL cell lines to profile circRNA expression and found several circRNAs, which have previously been implicated in cancer, indicating that circRNAs may play important roles in the pathogenesis of these diseases. Interestingly, we also detected circRNAs from genes involved in lymphomagenesis and the development of MM, including a circRNA derived from the *IKZF3* gene, which is not listed in circBase [50] and, to our knowledge, has not previously been reported.

We designed a panel of NanoString assays targeting the unique backsplicing junctions of 52 circRNAs based on the RNA-seq data, and showed that circRNA expression profiles can distinguish different B-cell malignancies. The specificity of our assays for circRNAs was addressed by treating samples with RNase R, and upon treatment we observed enrichment for all expressed circRNAs except circZNF91, whereas the linear mRNA targets that we used as reference genes in our study were degraded to various extents as expected. Regarding circZNF91, Sanger sequencing across the backsplicing junction was successful and it has previously been observed that particular circRNAs are sensitive to RNase R [25]. Because some circRNAs have been shown to be RNase R sensitive and the efficiency of RNase R treatments to remove linear RNA can vary [4], this data do not provide stringent proof that the assays solely target circular transcripts. However, the observed degree of enrichment in circRNA targets corresponds to what we expected and strongly supports that our NanoString assays are specific for circRNA detection. Furthermore, reporter and capture probes must bind immediately adjacent to one another to allow efficient binding of the hybridized complex to a streptavidin-coated slide cartridge and produce a target-specific signal. In the presence of a canonical linear transcript, the probes will bind with a gap between them, as well as in the opposite orientation required for proper stretching of the reporter molecule by electrophoresis and imaging [27].

We found that NanoString- and RNA-seq data did not correlate particularly well for all circRNAs, rather a systematic over- or underestimation of circRNA levels for some circRNAs was observed. Nevertheless, RT-qPCR data for these selected circRNAs generally correlated better with NanoString data. It may seem contradictory to use a method, which rely on RT and PCR for comparison. However, there are, to our knowledge, currently no other enzyme-free methods available for accurate circRNA quantification. Moreover, it has been shown that different polymerases introduce different artifacts/bias [53], thus, it was not expected that we would be able to reproduce the systematic bias observed between NanoString and RNA-seq when using RT-qPCR. Because variation can occur when using different bioinformatic algorithms for circRNA quantification in RNA-seq data [54], we also analyzed the data using another algorithm known as CIRI2 [30]. CIRI2 and find_circ correlated quite well but there were a few exceptions. For instance, find_circ did not detect the presence of circDDX21 in any of the samples, whereas CIRI2 did, and vice versa for circIKZF3. Nevertheless, most of the biases observed are likely of experimental nature relating to the RT and PCR amplification during library preparation for RNA-seq. We also found that most circRNAs detected by NanoString and not by RNA-seq were enriched in the RNase R experiments, whereas most of the circRNAs detected only by RNA-seq were not. Thus, our data indicate that the NanoString technology may be more accurate than RNA-seq for circRNA quantification and more sensitive and specific for the detection of circRNAs expressed at low levels.

Finally, we studied circRNA expression profiles in archival FFPE tissue samples from patients with various B-cell malignancies from which a fresh frozen sample was also available. The RNA extracted from these samples had RIN values between 1.5 and 2.5 indicating that the RNA was severely degraded. However, we observed very good correlations between the NanoString data obtained from these samples and the RNA samples from the paired fresh frozen tissues. The correlation was particularly good when the RNA from FFPE samples had a DV 200 value above 0.40. When performing hierarchical cluster analysis on the NanoString data from these and the paired samples, we observed a clear separation according to individual patients and not according to RNA quality. In contrast, the patient samples did not cluster according to diagnoses as could be expected from the data on the cell lines. This may be explained by the fact that patient samples have different background levels of RNA from normal cells as no micro- or macro dissections were performed in this study.

Currently, NanoString assays for circRNA quantification have to be custom made and RNA-seq is still the method of choice for the discovery of novel circRNAs. In addition, RNA-seq may also reveal internal splicing patterns of circRNAs when depleting linear RNA by an RNase R treatment followed by polyadenylation and poly(A) + RNA depletion [55].

In conclusion, we have shown that the NanoString technology is sensitive, specific and quantitatively accurate for the detection of circRNA in both high- and low-quality RNA samples from cell lines and samples from patients with B-cell malignancies. Because this technology does not rely on RT and PCR amplification, which are known to create artifacts, it has the potential to become the gold standard method for circRNA quantification.

Supplementary Information accompanies the paper on the Laboratory Investigation website (http://www.nature.com/labinvest/).

## Electronic supplementary material

Supplementary information
