# Supplementary material for: Enzyme-free digital counting of endogenous circular RNA molecules in B-cell malignancies
Source: Lab Invest. 2018 Aug 7;98(12):1657–69. doi: 10.1038/s41374-018-0108-6 (PMC6265260; doi:10.1038/s41374-018-0108-6)
Supplement: Supplementary file 1 — Supplementary information [file 41374_2018_108_MOESM1_ESM.docx]

**Supplementary Figures and Tables**

**
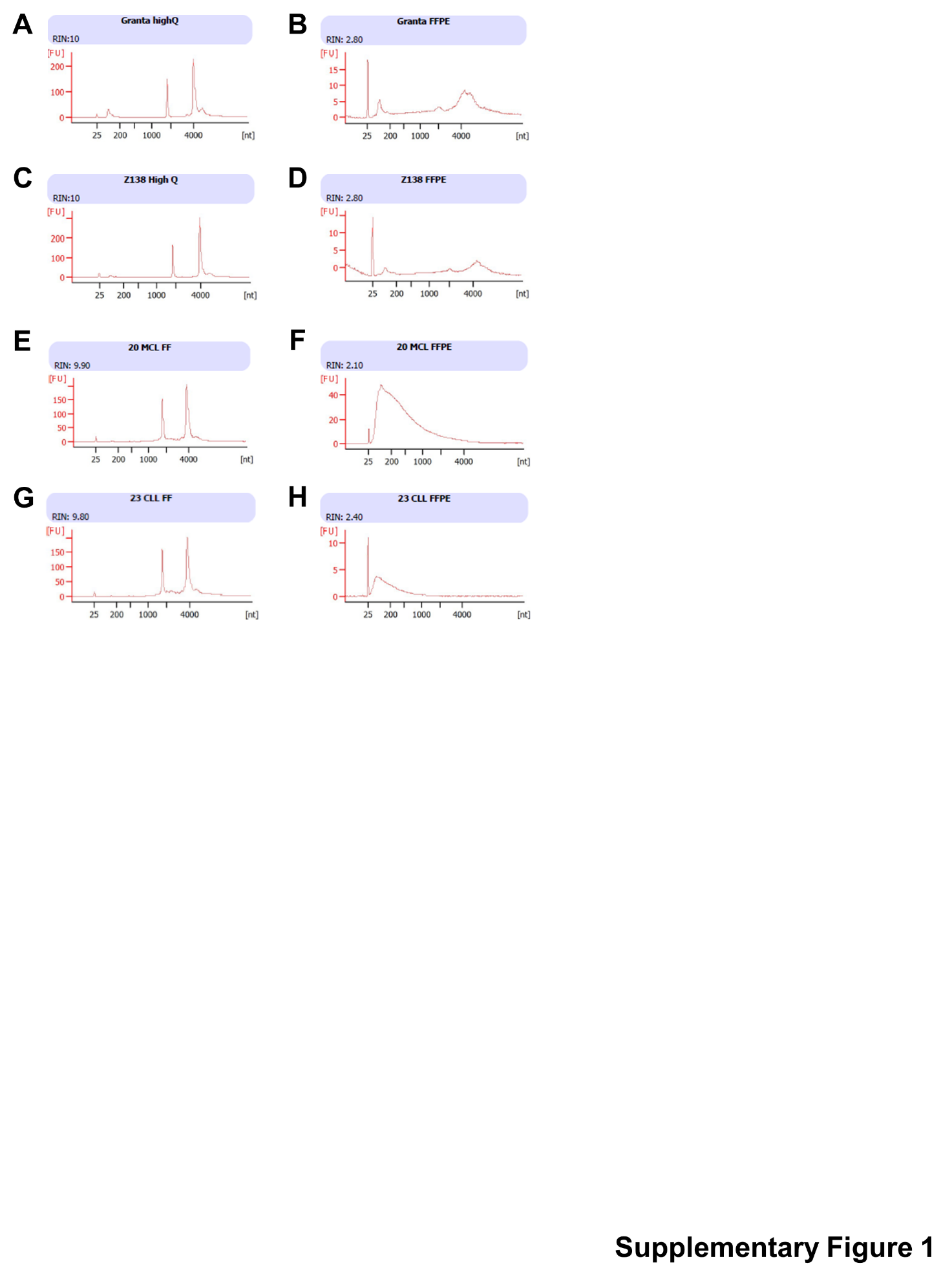
**

**Supplementary Figure 1. Assessment of RNA quality in paired fresh-frozen (FF) and formalin-fixated, paraffin-embedded (FFPE) cell lines and tissue samples. (A-H)** Representative bioanalyzer profiles for Granta-519 (A-B), Z138 (C-D), patient 20 (E-F) and patient 23 (G-H). Displayed are electropherograms and corresponding RIN-values of RNA from paired high quality (Cell line) or FF (patient) (left panels) and FFPE (Cell line or patient) samples (right panels).


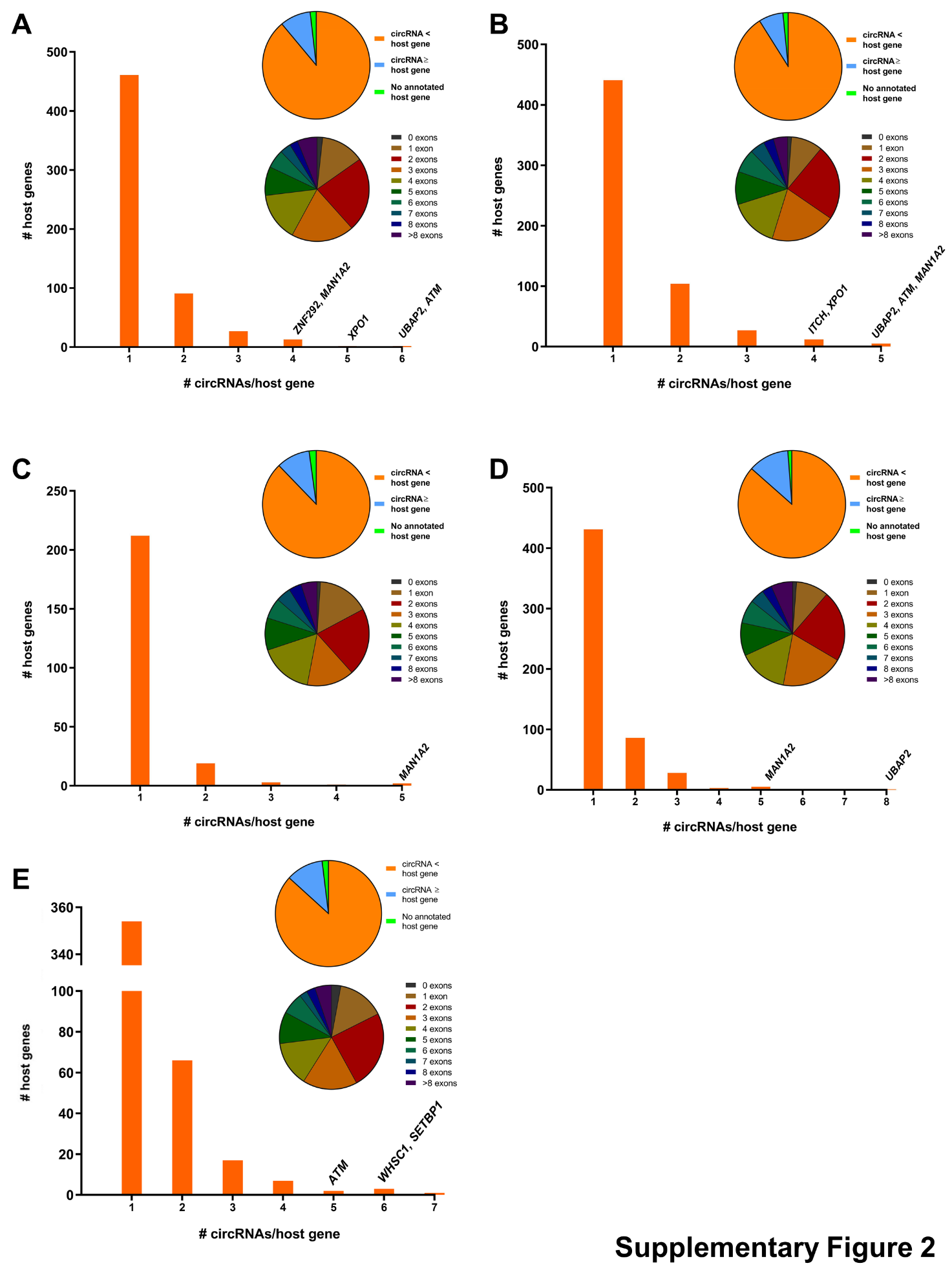


**Supplementary Figure 2. Bioinformatic characterization of the circRNAs detected in the MM and MCL cell lines.** The number of circRNA per host gene is summarized in barplots for RNA-seq data from the cell lines REC-1 (A), Granta-519 (B), UPN-2 (C), Z138 (D), and NCI-H929 (E). Host genes from which multiple circRNAs derive are denoted. Moreover, for each cell line, Pie charts show the distribution of circRNAs with higher or lower expression than their respective host genes (top) and the distribution of exon-composition within circRNAs (bottom).

**
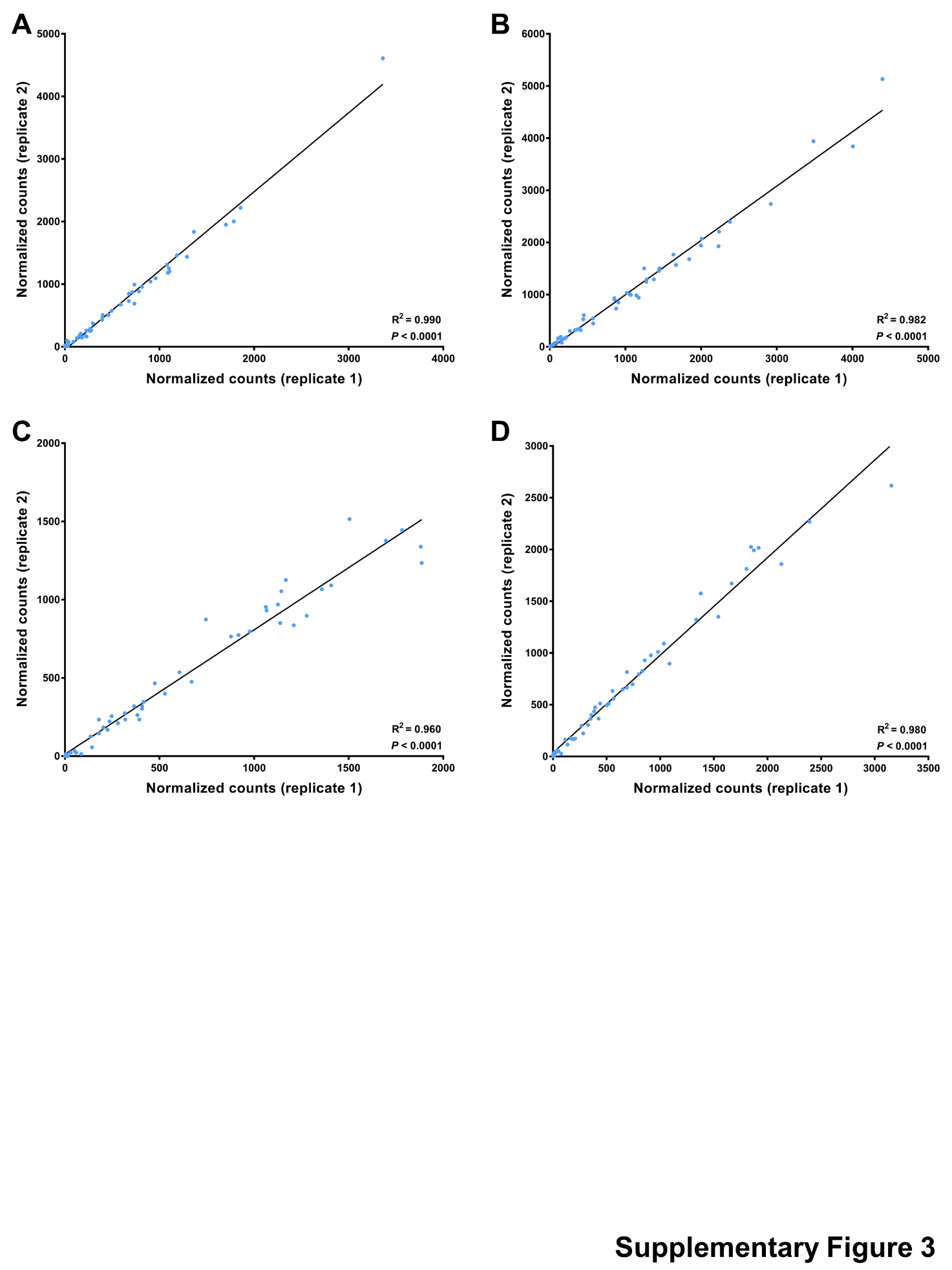
**

**Supplementary Figure 3.** **The NanoString technology provides reproducible data for circRNA quantification.** Technical replicates of RNA samples of high quality (A and C) and RNA of low quality from FFPE cells (B and D) from the cell lines REC-1 (A-B) and Granta-519 (C-D) were analyzed twice on different days.


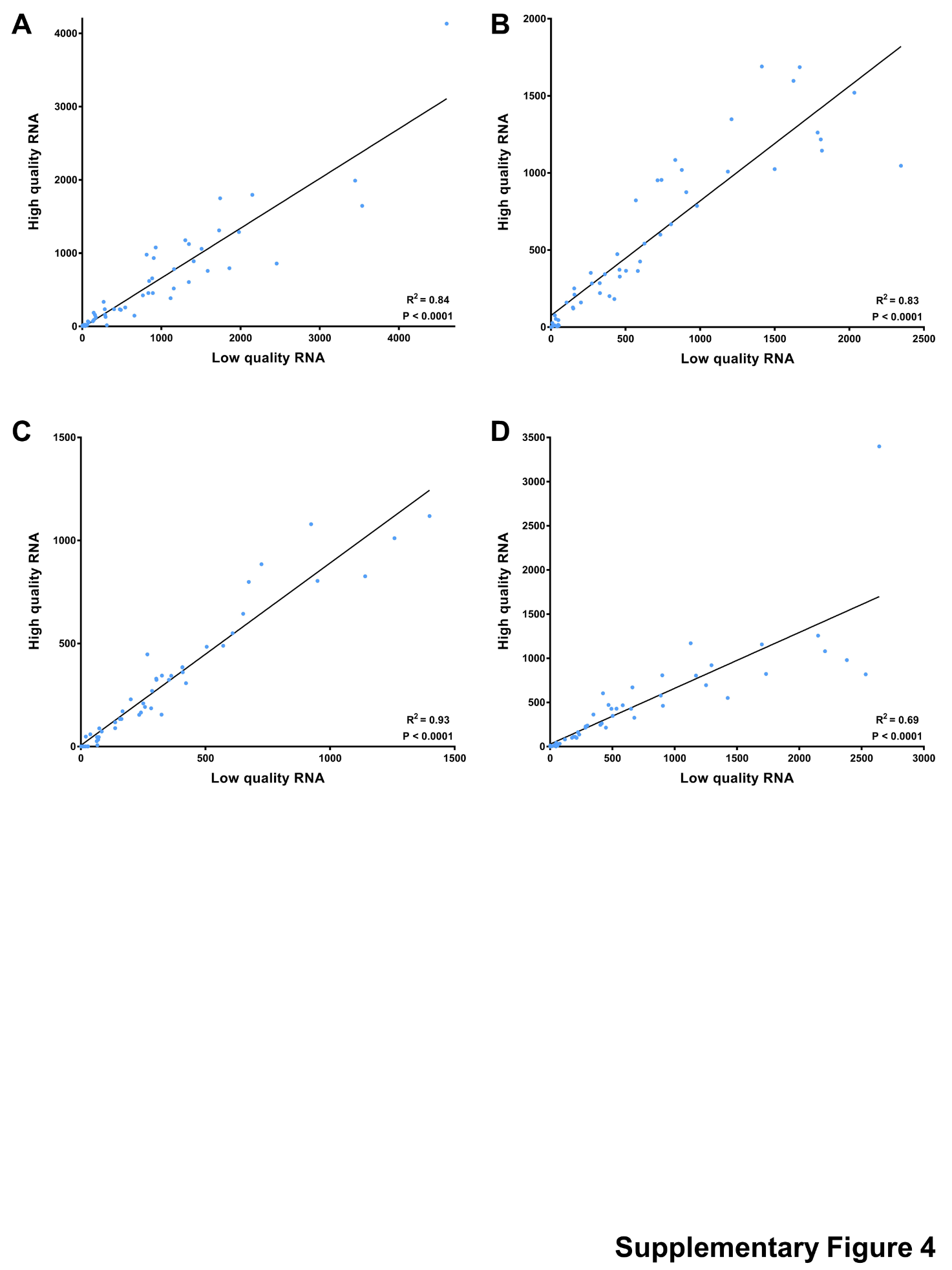


**Supplementary Figure 4. Correlation between NanoString data from high- and low quality RNA from cell lines. (A-D)** RNA was either isolated as high Quality RNA or as low quality RNA from FFPE MCL cell lines. Correlation between NanoString quantification (normalized counts) of high quality RNA and RNA from FFPE samples from REC-1 (A), Granta-519 (B), UPN-2 (C), and Z138 (D) is shown with linear regression statistics.

**
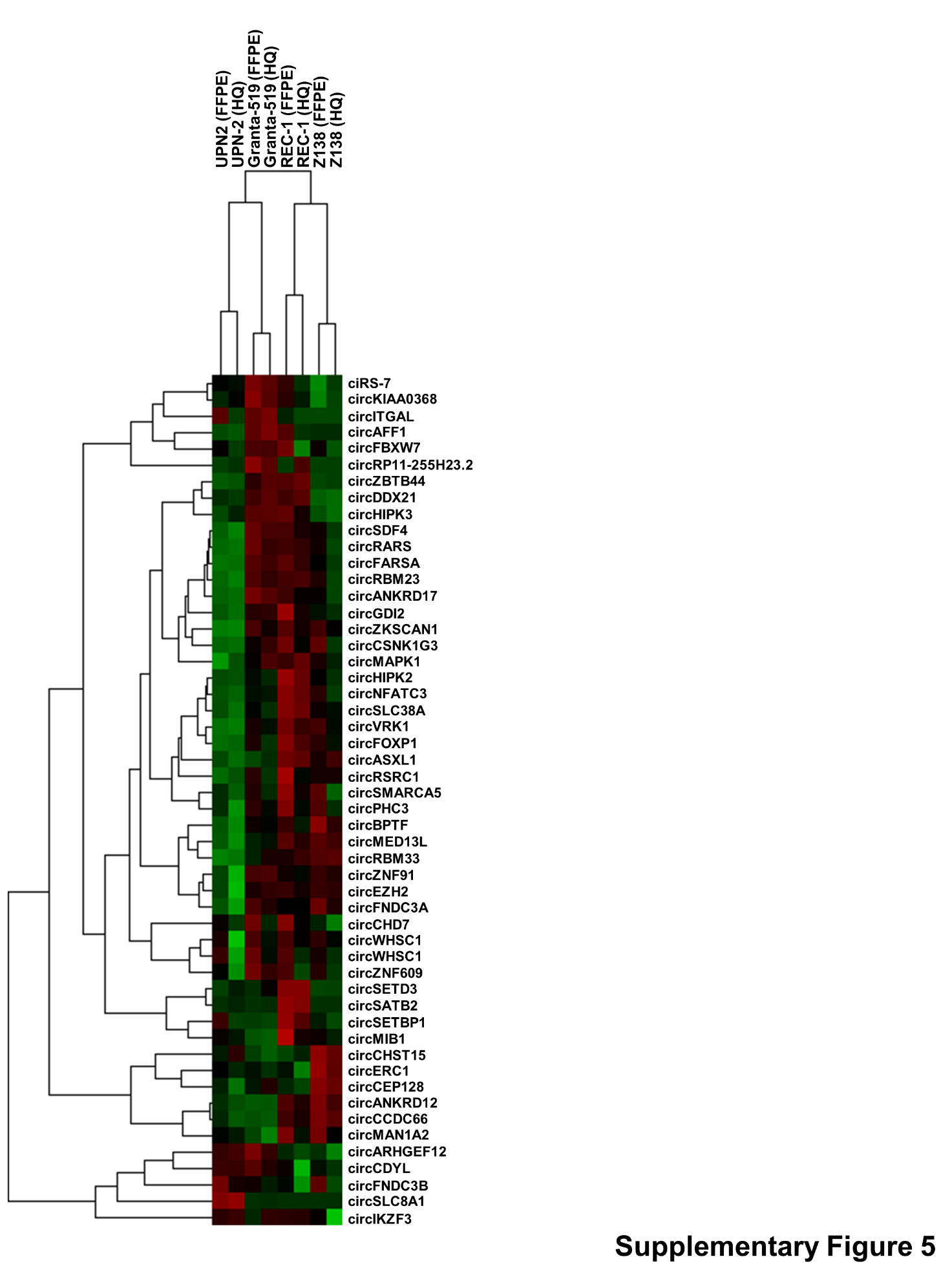
**

**Supplementary Figure 5. Heat map and hierarchical cluster analysis of NanoString data from high- and low-quality RNA from cell lines.** The data from low-quality RNA, isolated from formalin-fixed and paraffin-embedded MCL cell lines, were compared with data from high-quality RNA from the same cell lines.


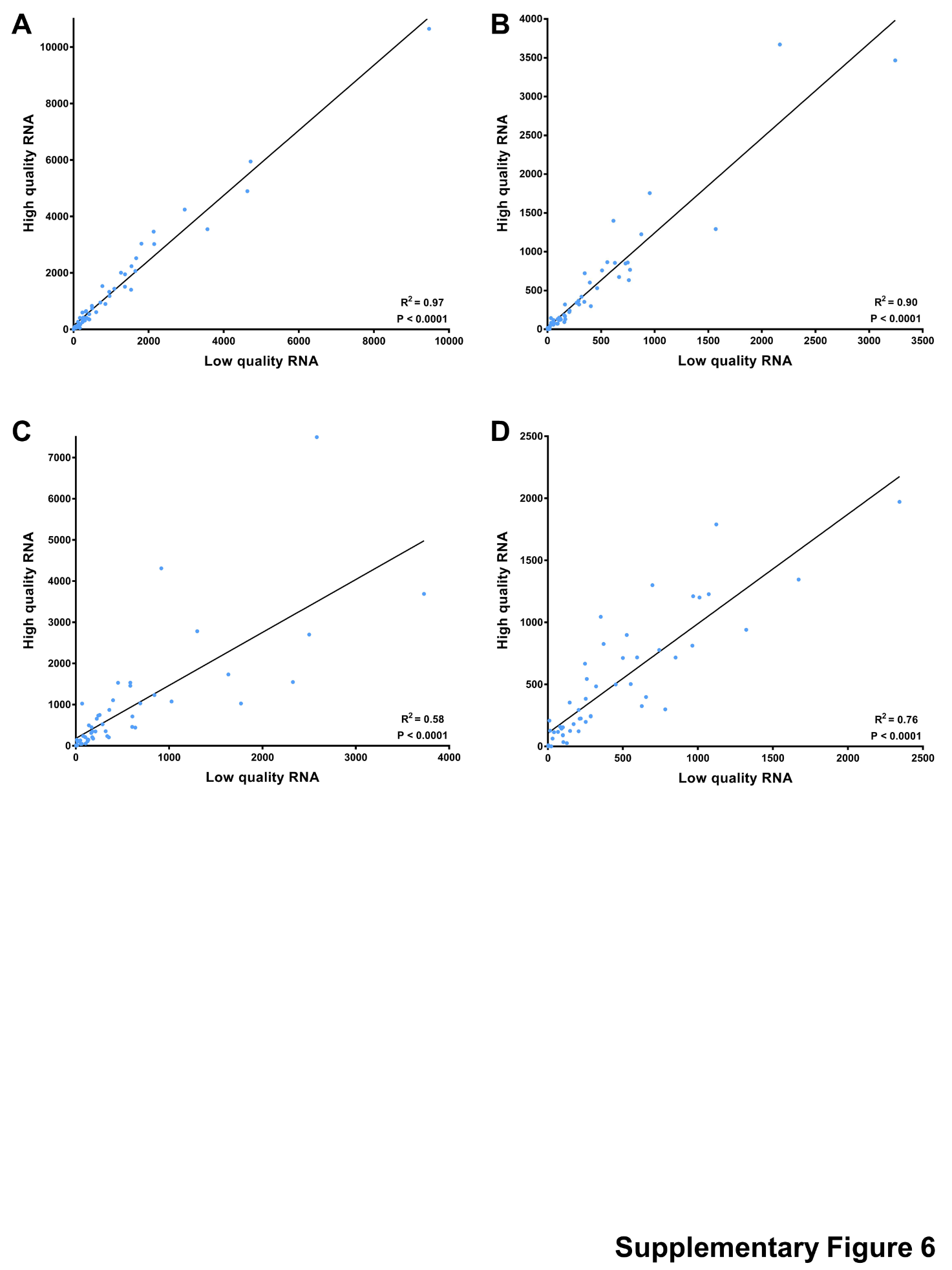


**Supplementary Figure 6. Correlation between NanoString data from high- and low quality RNA from paired fresh frozen and FFPE patient samples. (A-D)** Correlation between representative NanoString quantifications (normalized counts) of FF and FFPE RNA from Patient 15, DV 200 > 0.40 (A), Patient 10, DV 200 > 0.40 (B), Patient 13, DV 200 < 0.33 (C), Patient 8, DV 200 < 0.33 (D, one outlier removed).

**
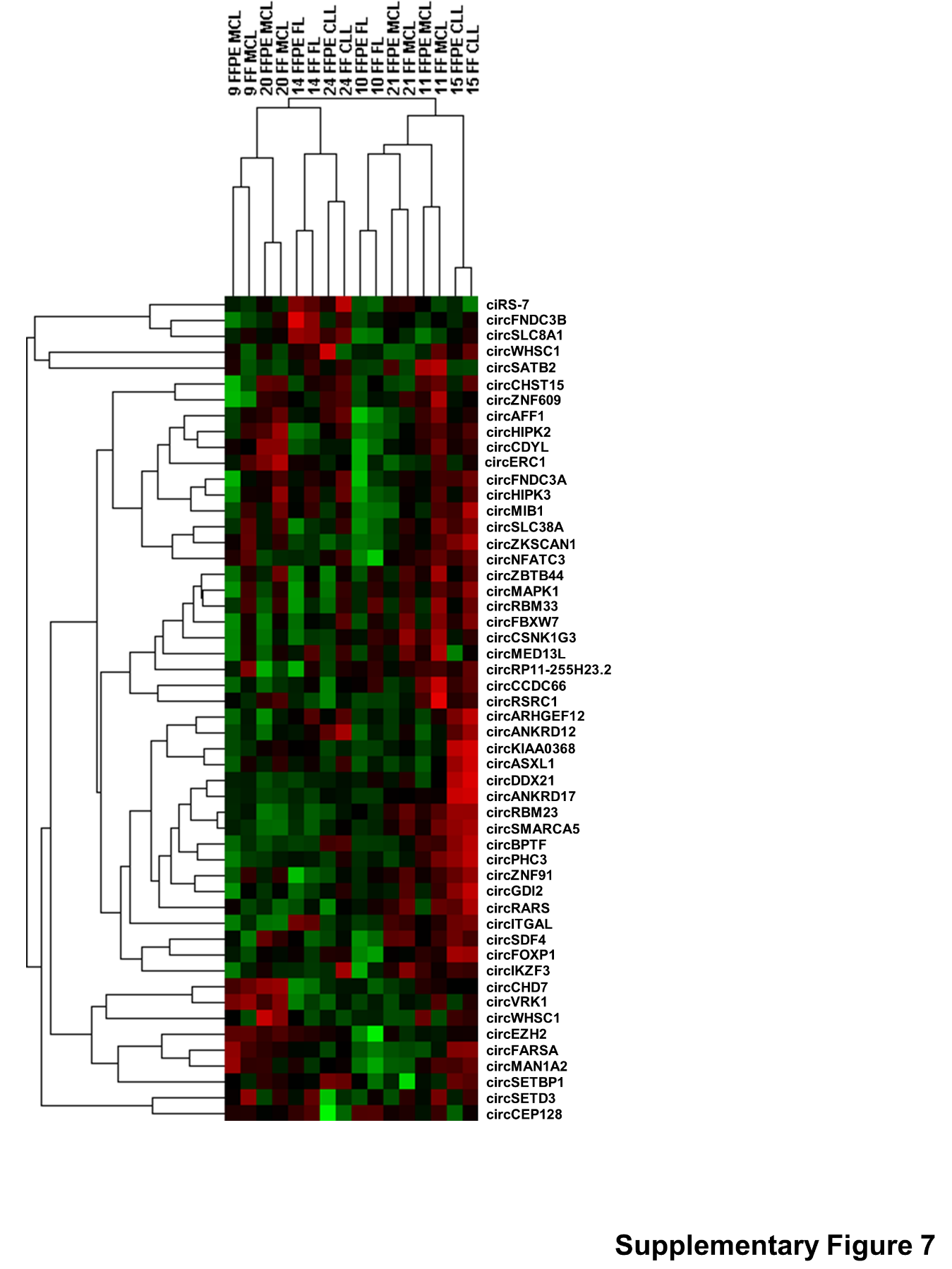
**

**Supplementary Figure 7. Heat map and hierarchical cluster analysis of NanoString data from high- and low quality RNA from paired fresh frozen and FFPE patient samples.** Sample pairs for which the DV 200 value of the RNA from the FFPE sample was above 0.40 were analyzed. For the patients, diagnosis and sample origin are indicated. FFPE; Formalin-Fixed Paraffin-Embedded, FF; Fresh Frozen.


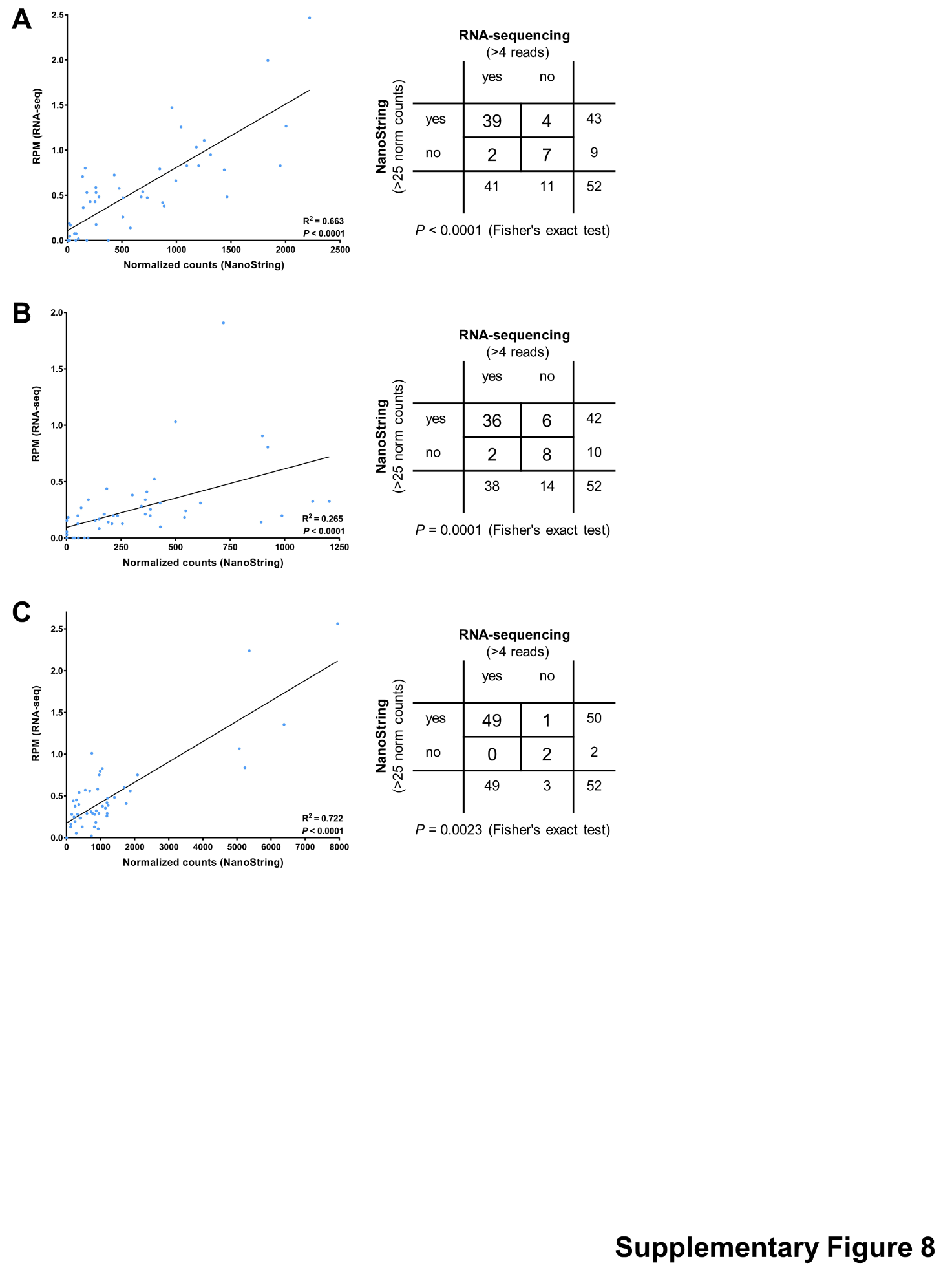


**Supplementary Figure 8. Comparison of circRNA quantification by NanoString and RNA-seq for the cell lines where no RNase R experiments were performed. (A-C)** Continuous (left panels) and dichotomous (right panels) comparison of RNA-seq and NanoString quantification (as in Fig. 5) on RNA from the cell lines REC-1 (A), UPN-2 (B), and NCI-H929 (C). circASXL1 was an extreme outlier and removed from the analyses.

**
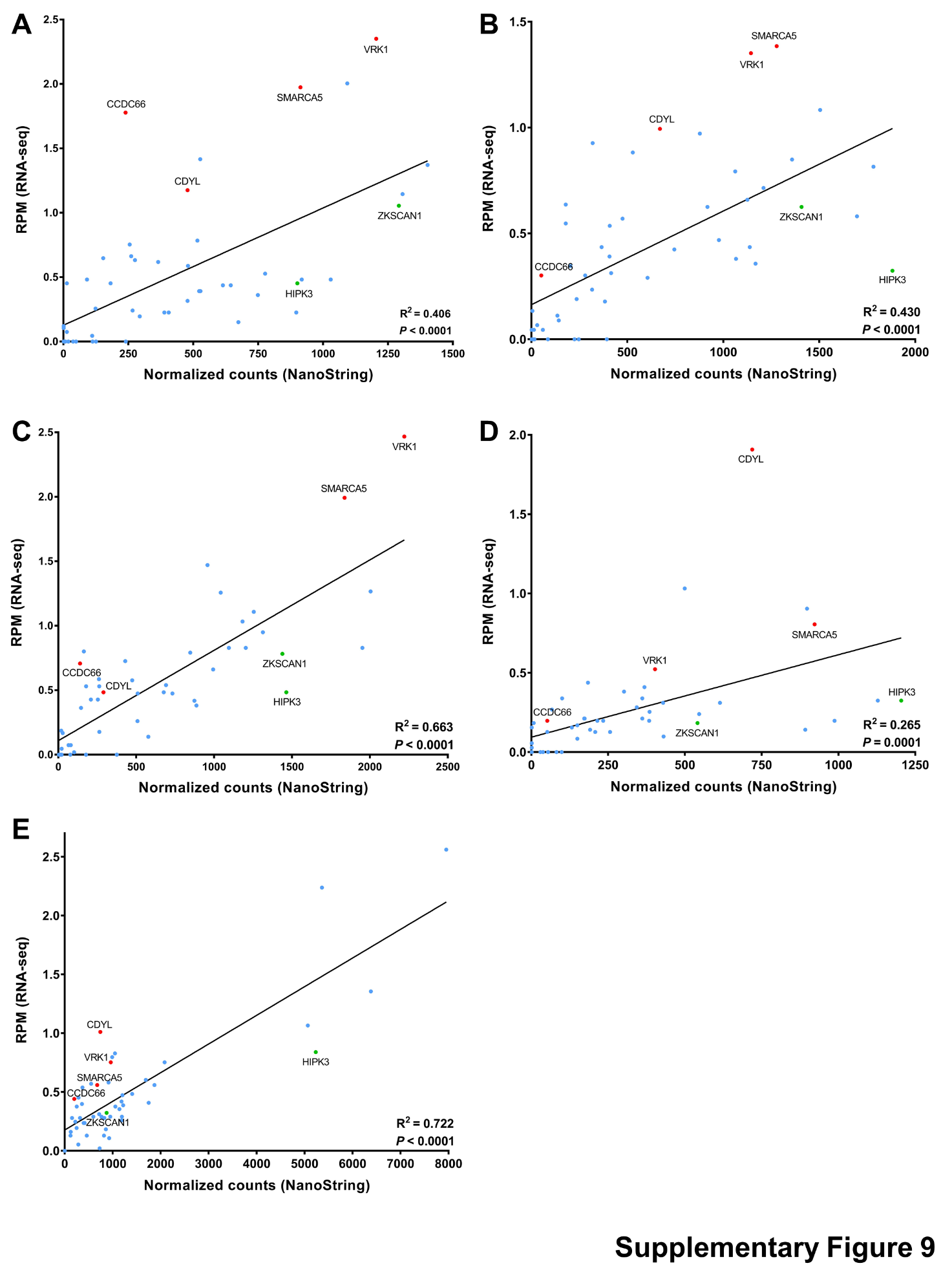
**

**Supplementary Figure 9. Consistent bias between NanoString and RNA-seq quantification. (A-E)** Comparison between RNA-seq and NanoString (as in Figure 5 and Supplementary Fig. 8) with **s**pecific circRNAs (indicated) either systematically overestimated (circCCDC66, circCDYL, circVRK1, circSMARCA5) or underestimated (circZKSCAN1, circHIPK3) in RNA-seq data from the cell lines Z138 (A), Granta-519 (B), REC-1 (C), UPN-2 (D), and NCI-H929 (E).


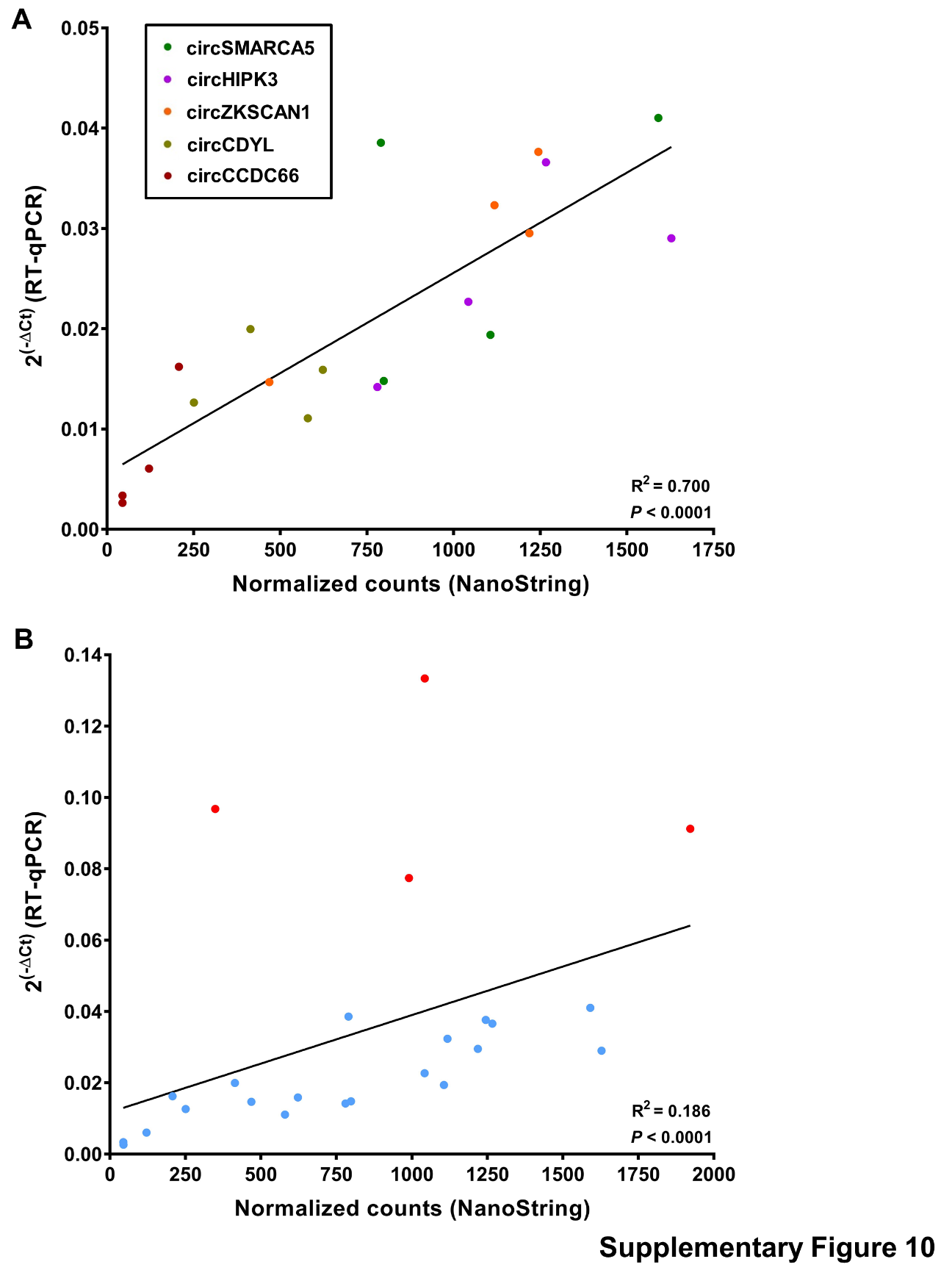


**Supplementary Figure 10. Comparison between NanoString and RT-qPCR data. (A)** Correlation between NanoString- and RT-qPCR data on five different circRNAs (color-coded as denoted). Each dot corresponds to data obtained in one of the four MCL cell lines. **(B)** As in A, but including the quantification of circVRK1 (red).


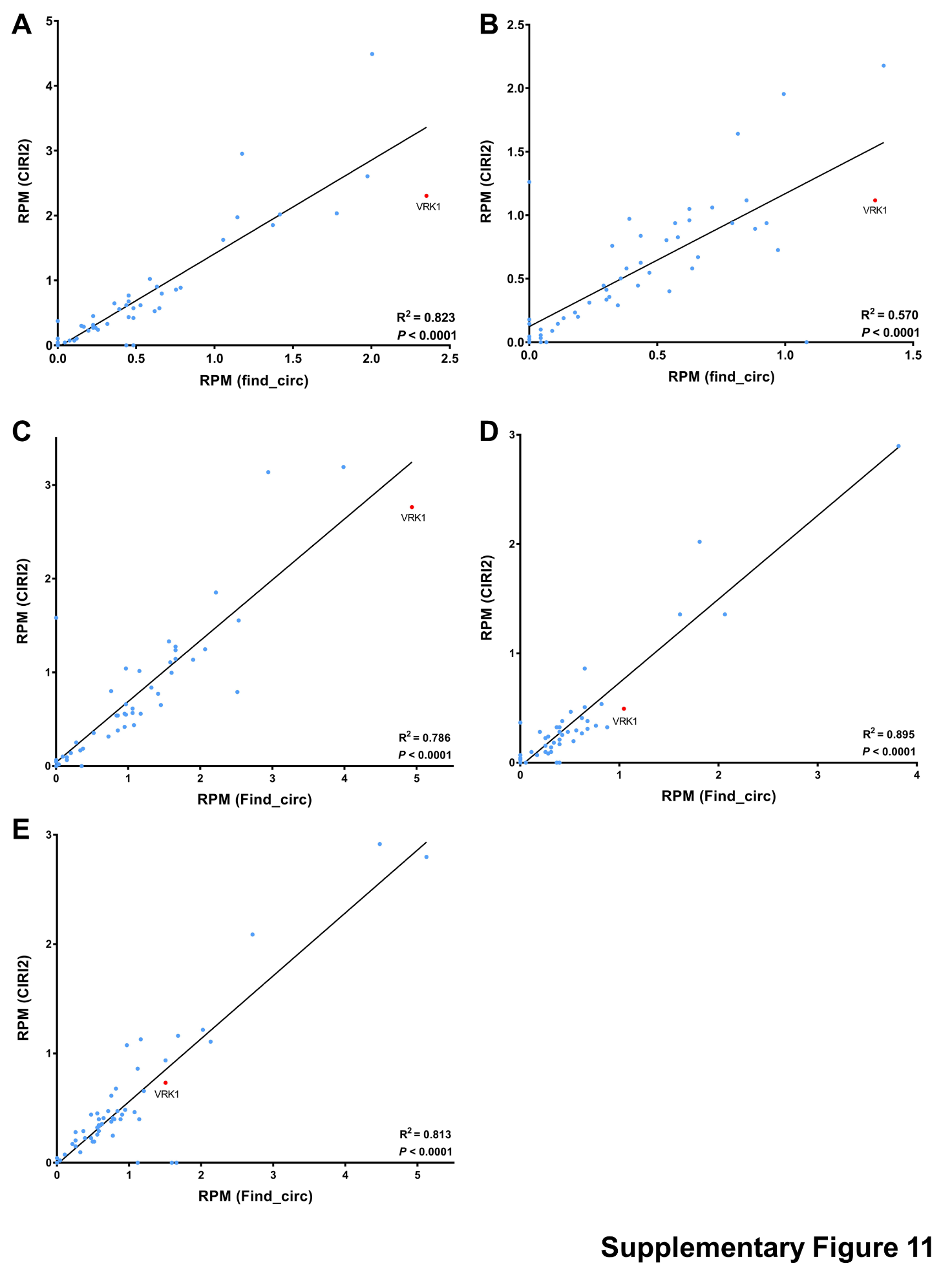


**Supplementary Figure 11. Comparison between two different bioinformatics algorithms for circRNA quantification in RNA-seq data.** Scatterplot comparing circRNA quantification by find_circ and CIRI2 using RNA-seq from the cell lines Z138 (A), Granta-519 (B), REC-1 (C), UPN-2 (D), and NCI-H929 (E). circVRK1, which is systematically skewed between find_circ and CIRI2 quantification, is denoted in red.


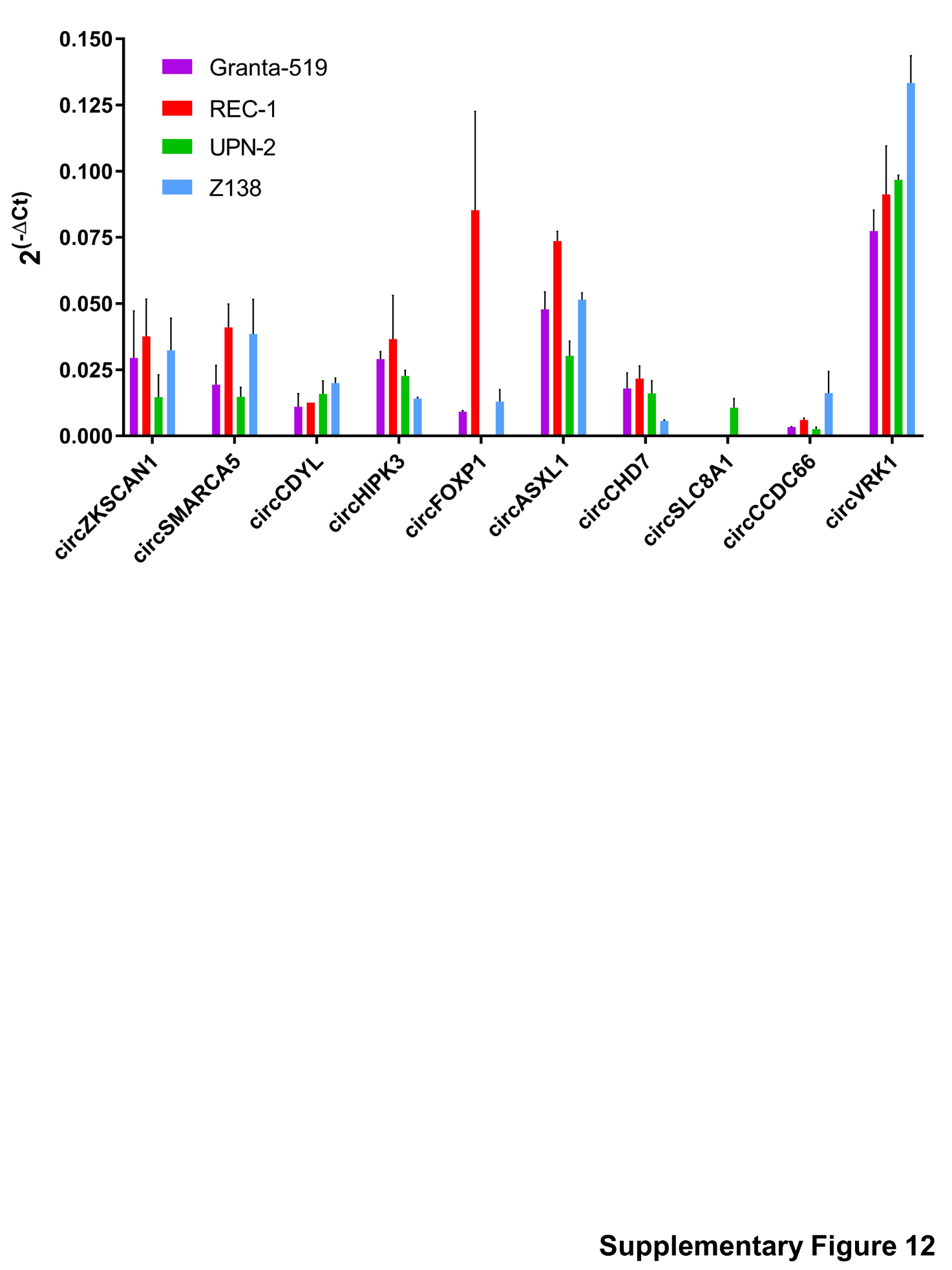


**Supplementary Figure 12. RT-qPCR data for ten circRNAs in the four MCL cell lines.** The data were normalized using the same four reference genes as for the NanoString data. Error bars represent standard deviation.


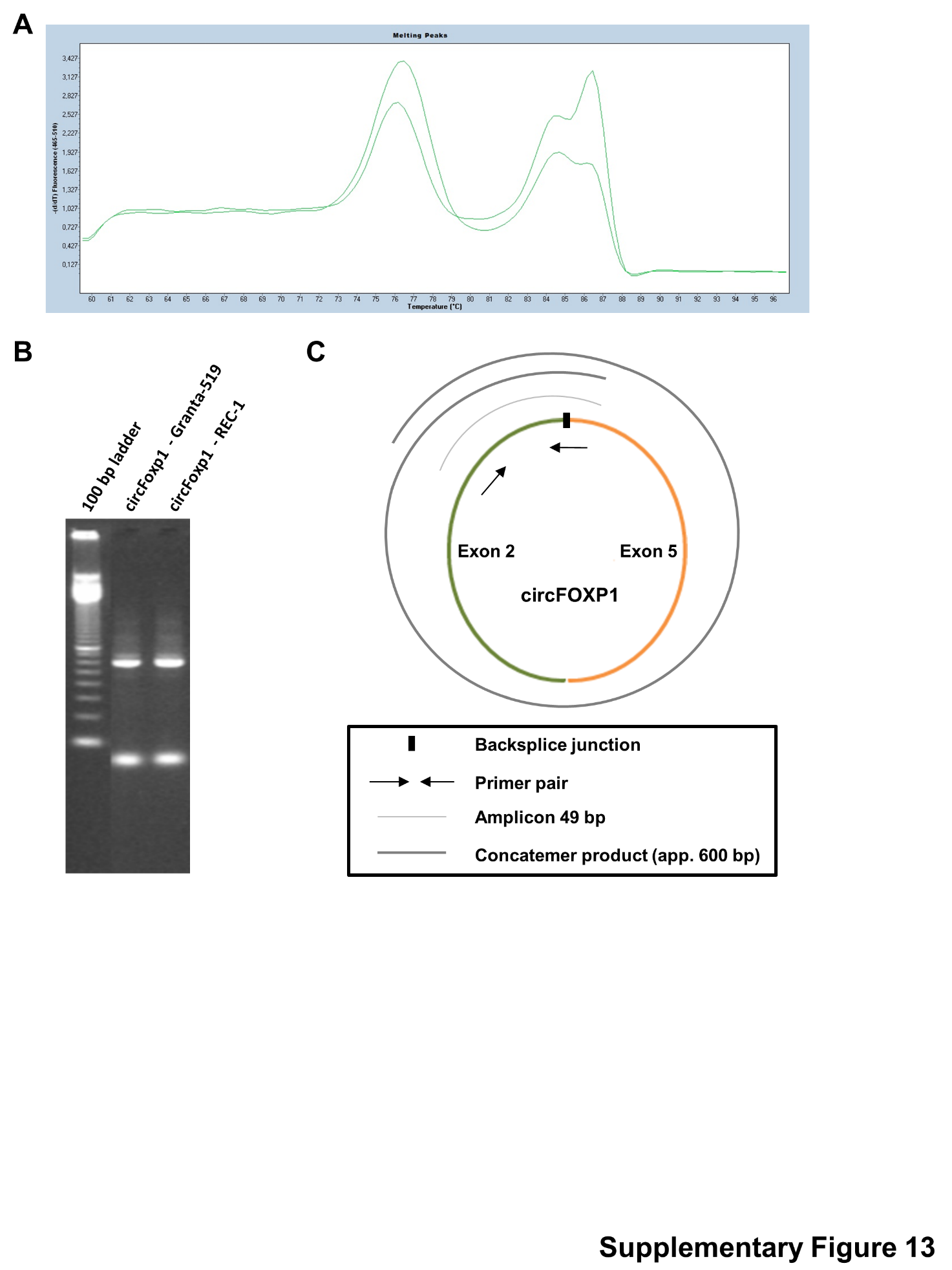


**Supplementary Figure 13. Concatemer formation detected by melting curve analyses and gel electrophoresis. (A)** Melting curves for the circFOXP1 RT-qPCR assay using high quality RNA from the cell lines Granta-519 and REC-1 as template. **(B)** Agarose gel electrophoresis of circFOXP1 RT-PCR products from (A) showing ~50bp- and ~600 bp amplicon. **(C)** Figure displaying the concept of concatemer formation by ‘rolling circle’ RT amplification.


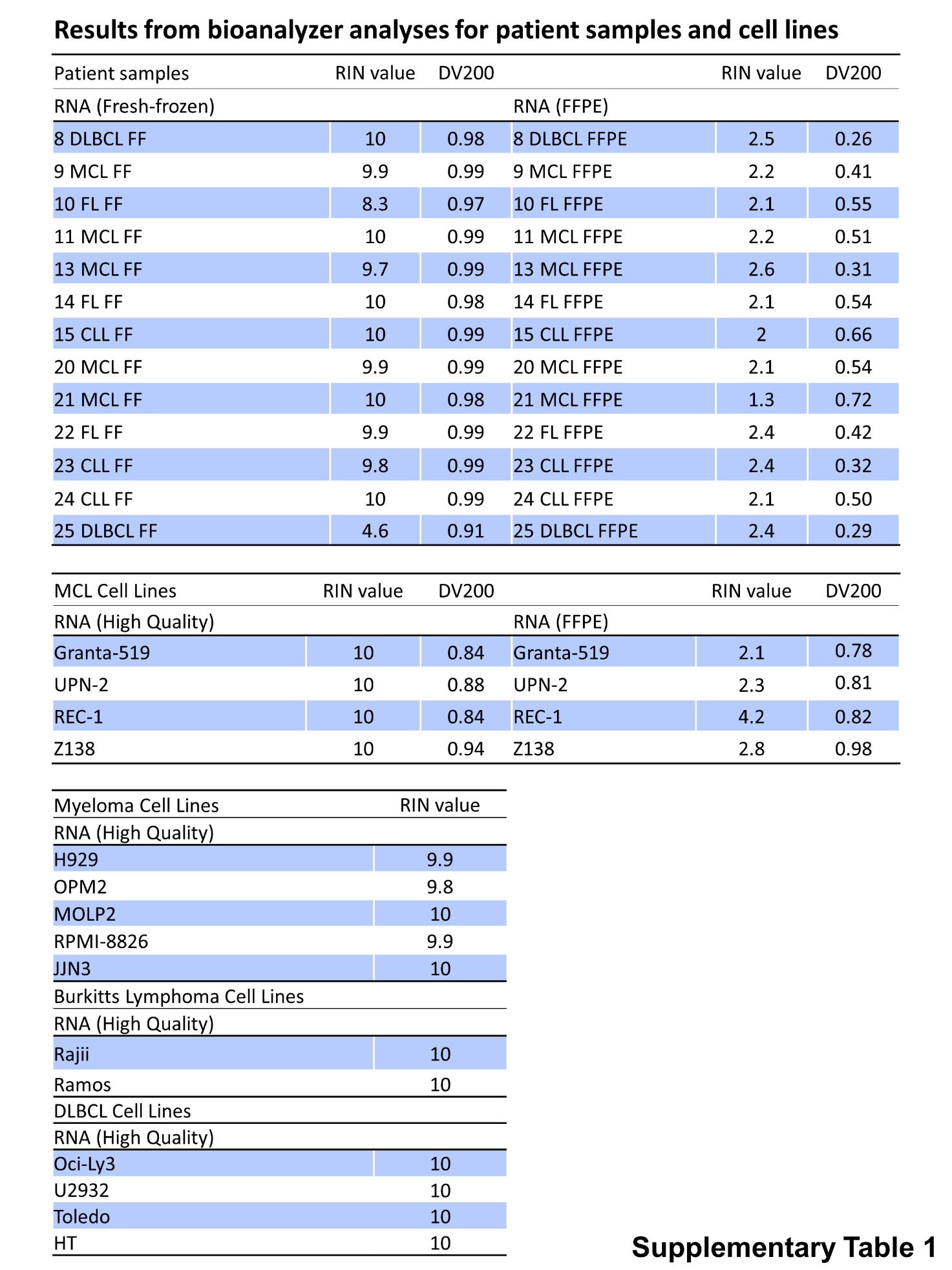


**Supplementary Table 1. Bioanalyzer results. RIN values (RNA integrity number) and DV200 values (percentage of RNA fragments above 200 nucleotides) are shown for patient samples and cell lines.**

**
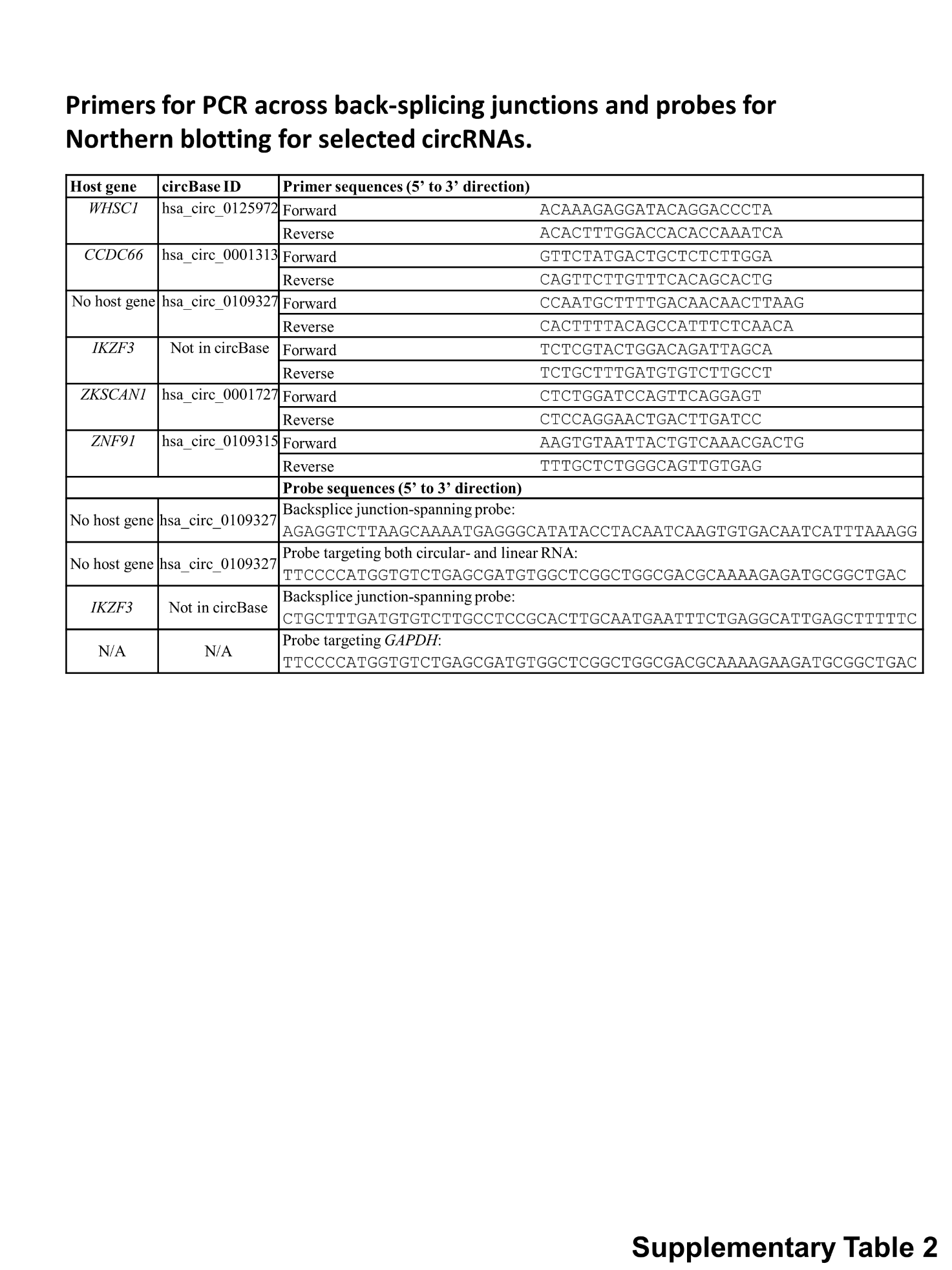
**

**Supplementary Table 2. Primers for PCR across backsplicing junctions and probes for Northern blotting of selected circRNAs.**

**
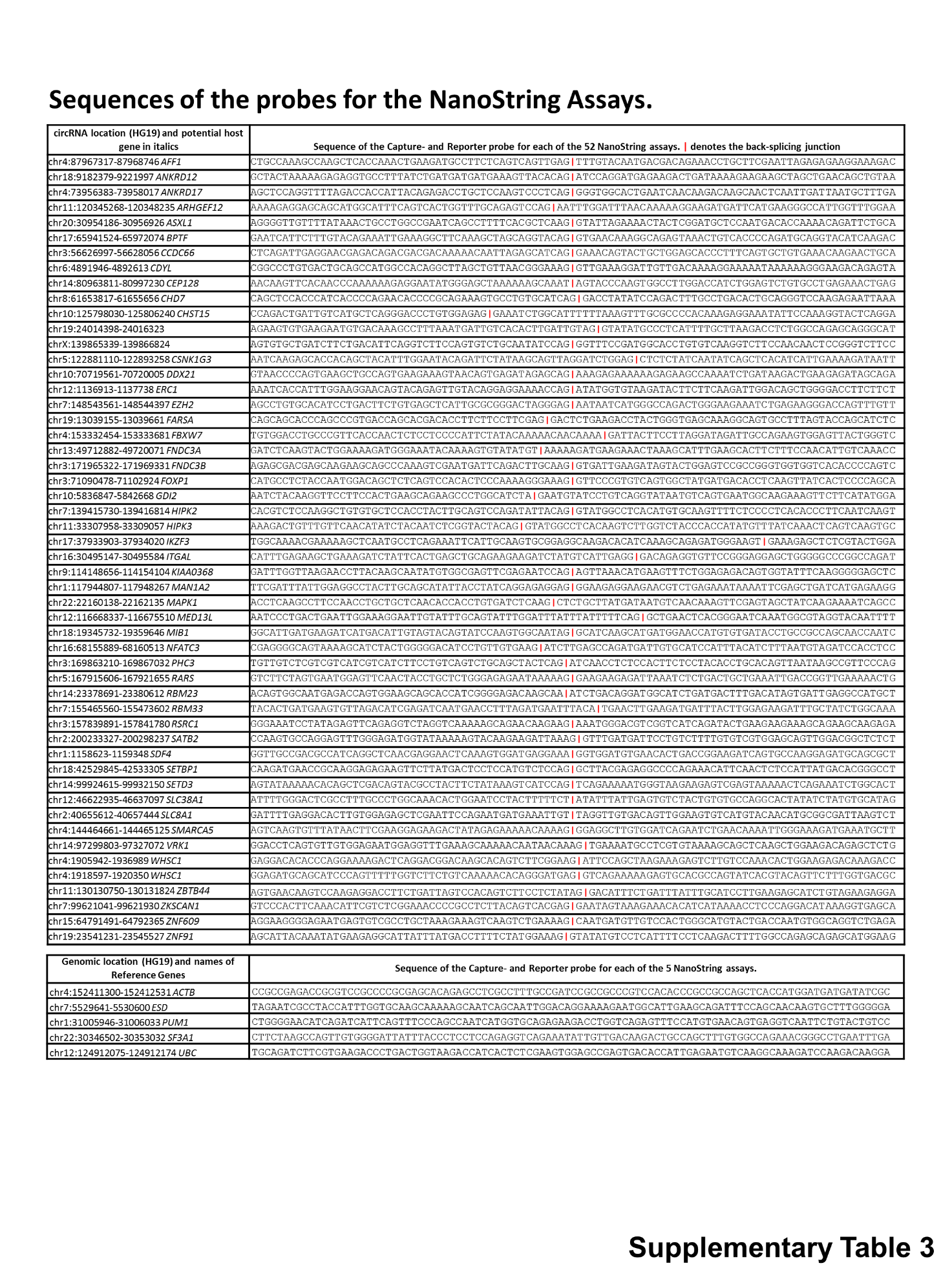
**

**Supplementary Table 3. Sequences for design of the probes for the NanoString Assays targeting circRNA candidates or reference genes.**

**
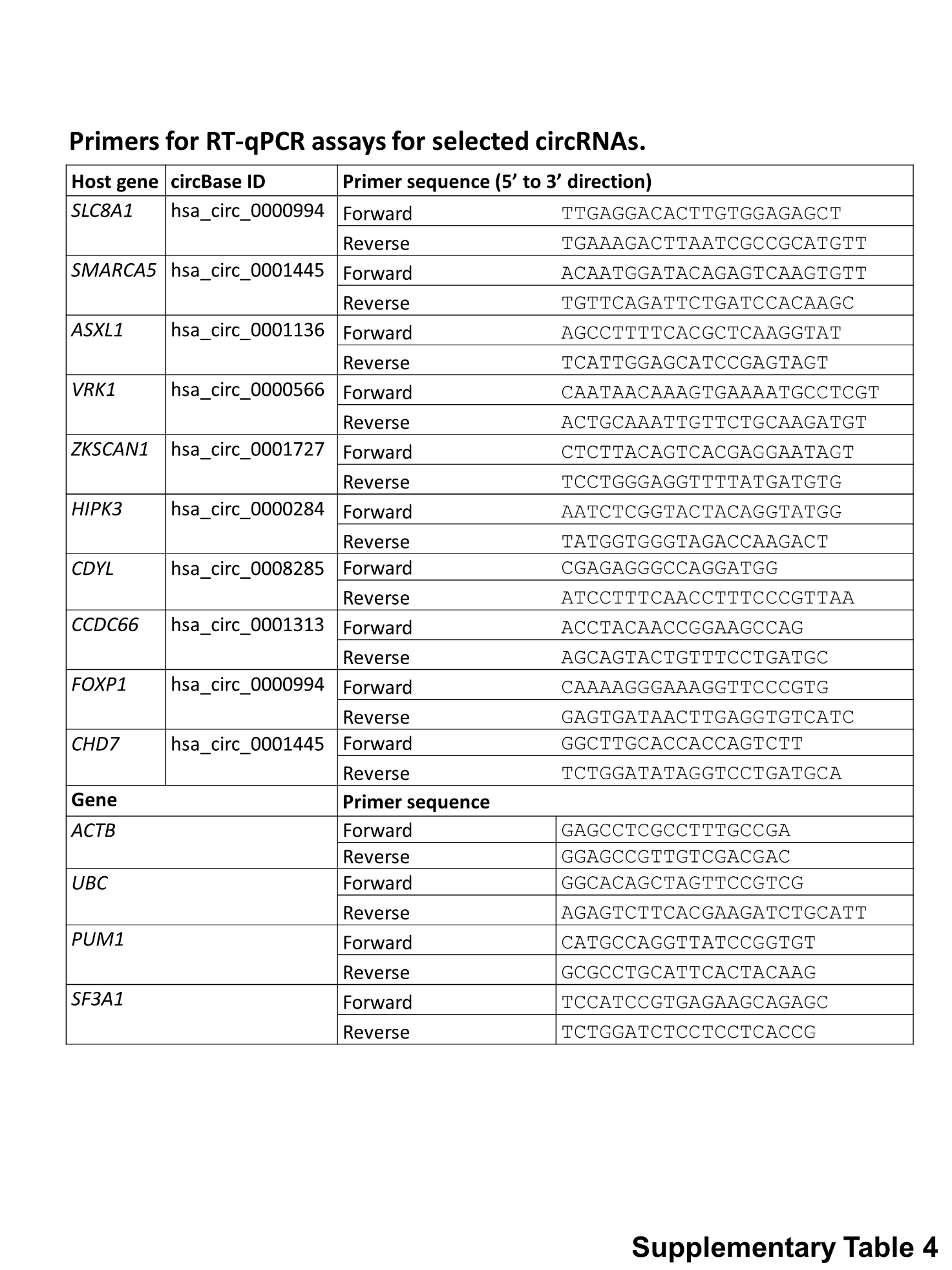
**

**Supplementary Table 4. Primers for RT-qPCR assays for selected circRNAs and reference genes.**

**
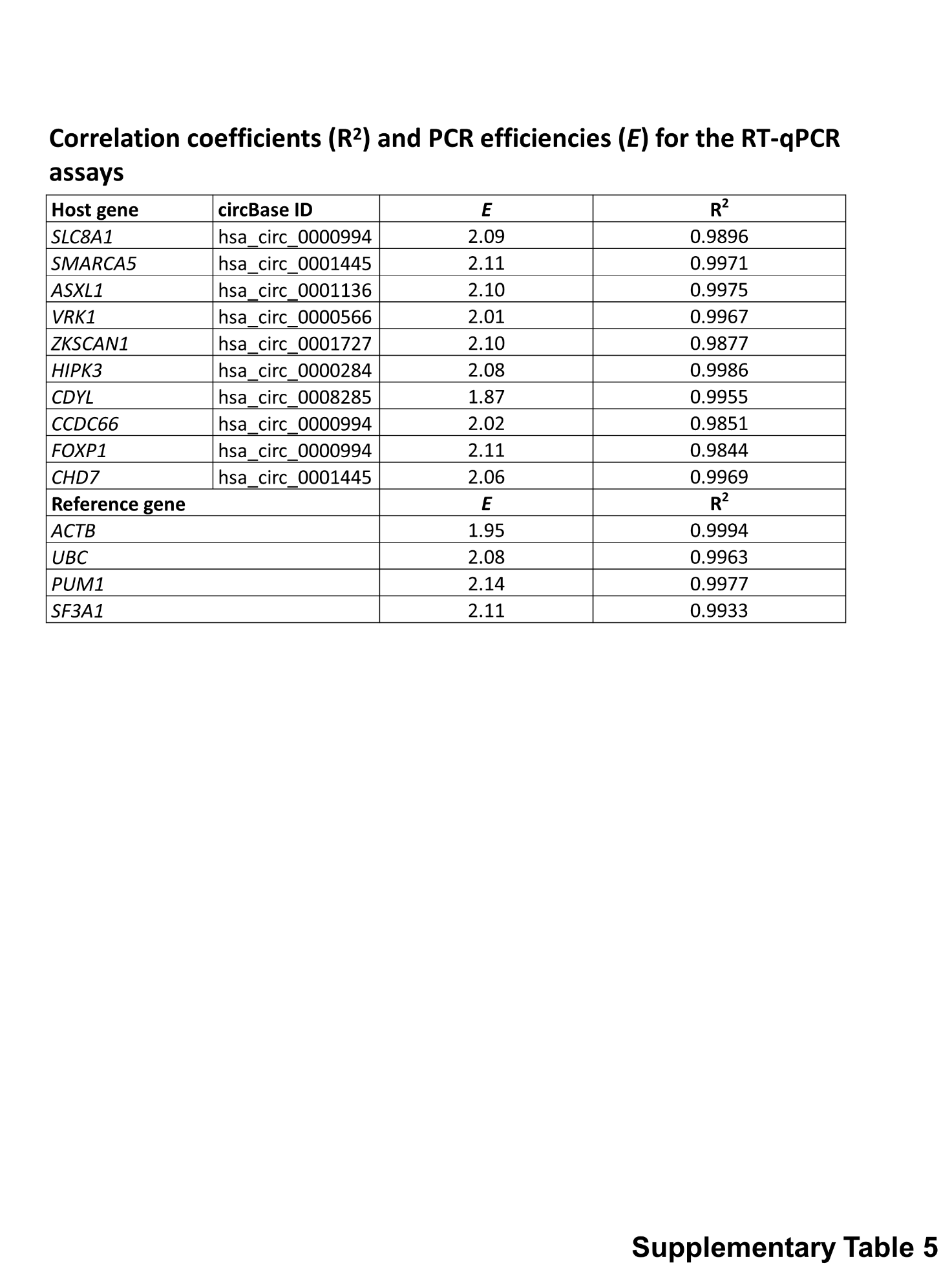
**

**Supplementary Table 5. Correlation coefficients (R^2^) and PCR efficiencies (*E*) for the RT-qPCR assays.**
